# Developments in asthma incidence and prevalence in Alberta between 1995 and 2015

**DOI:** 10.1186/s13223-020-00485-3

**Published:** 2020-10-09

**Authors:** Ana-Maria Bosonea, Heather Sharpe, Ting Wang, Jeffrey A. Bakal, A. Dean Befus, Lawrence W. Svenson, Harissios Vliagoftis

**Affiliations:** 1 Division of Pulmonary Medicine, Department of Medicine, University of Alberta, 11350 83rd Ave, 3-134b Clinical Sciences Building, Edmonton, AB T6G 2G3 Canada; 2 Clinical Allergy and Immunology, University of British Columbia, Vancouver, Canada; 3 Respiratory Health Strategic Clinical Network (RHSCN), Alberta Health Services (AHS), Edmonton, Canada; 4 Provincial Research Data Services-Alberta Health Services, Edmonton, Canada; 5 Alberta Respiratory Centre, University of Alberta, Edmonton, Canada; 6 Analytics & Performance Reporting Branch, Alberta Health, Edmonton, Canada; 7 Division of Preventive Medicine, University of Alberta, Edmonton, Canada; 8 School of Public Health, University of Alberta, Edmonton, Canada; 9 Department of Community Health Sciences, University of Calgary, Calgary, Canada

**Keywords:** Asthma, Prevalence, Alberta, Incidence, Epidemiology

## Abstract

**Background:**

Asthma is a chronic respiratory disease characterized by reversible bronchoconstriction and airway inflammation. According to Statistics Canada in 2014, 8.1% of Canadians aged 12 and older reported having asthma diagnosed by a health care professional. Therefore, in 2014 there were an estimated 274,661 persons with asthma in Alberta. Most epidemiological studies estimate prevalence and incidence using survey-based data, which has limitations. The Ontario Asthma Surveillance Information System (OASIS) group has developed and validated an algorithm for epidemiologic asthma studies using provincial health databases. In Alberta, there are some studies using provincial databases, but most are restricted to emergency department visits and do not represent the entire asthma population. Using the validated asthma definition for epidemiologic studies, we performed an analysis of the Alberta Health administrative databases to investigate and report province-wide asthma prevalence, incidence and mortality in Alberta from 1995 to 2015.

**Methods:**

Data from administrative databases, provided by Alberta Health, was analyzed to determine age and sex specific prevalence, incidence and mortality of the asthma population. The population cohort was all individuals residing in the province of Alberta, ages 0 to 99 from 1995–2015. Kendall’s Tau coefficient test was used to ascertain whether the observed trends were statistically significant.

**Results:**

Between 1995 and 2015, the age-standardized incidence of asthma decreased by more than 50% in both males and females. Prevalence, however, increased threefold over the 20 years (for both genders) from 3.9 to 12.3% (Tau = 1.00, p < 0.0001) in females and from 3.5 to 11.6% (Tau = 1.00, p < 0.0001) in males. Thus, in 2015 there were 496,927 people with asthma in Alberta. All-cause mortality in the asthma population decreased over time, in both females (Tau = − 0.71, p < 0.0001) and males (Tau = − 0.69, p = 0.0001). For the last several years, all-cause mortality was higher in those with asthma. There were ~ 7 deaths/1000 in the population with asthma versus ~ 5 deaths/1000 in those without asthma.

**Conclusions:**

The incidence of asthma decreased in both females and males while prevalence continued to increase, although at a slower rate than previously. All-cause mortality in asthma patients was higher than in those without asthma, but both decreased over time.

## Background

Asthma is a chronic respiratory disease characterized by reversible bronchoconstriction and airway inflammation. In 2018, an estimated 339 million people world-wide had asthma [1]. In the United States, the prevalence of asthma increased from 2001 to 2010 and then seemed to stabilize at 8.2% of the population in 2015 [2, 3]. According to Statistics Canada survey data in 2014, 8.1% of Canadians aged 12 and older, 2.4 million people, reported having asthma diagnosed by a health professional. This has not changed significantly since 2001. Thus, there were an estimated 274,661 people living with asthma in Alberta in 2014 [4].

The above data and most epidemiological studies of asthma estimate the prevalence and incidence using survey-based data, which has a high rate of differential recall bias and may overlook portions of a population [5, 6]. In contrast, The Ontario Asthma Surveillance Information System (OASIS) group uses health databases to create a population-based longitudinal surveillance system that identifies and tracks individuals living with asthma in the province of Ontario. OASIS has validated an algorithm for the definition of asthma, when using healthcare databases, as: two or more ambulatory care visits in 2 consecutive years or one or more hospitalization(s) for asthma. This algorithm had a sensitivity of 83.8% and a specificity of 76.5% and has been used for epidemiological investigations in both adults and children [7, 8]. In 2010, Gershon et al*.* reported that the prevalence of asthma in Ontario increased from 8.5% in 1996 to 13.3% in 2005. The increase in prevalence was greatest in adolescents and young adults compared with other age groups and greater in males than females [9].

In Alberta, there are several high-quality studies using provincial databases [10–12], reporting on the epidemiology of emergency department (ED) visits for asthma, including a publication from Rosychuk et al. showing that ED visits for asthma declined from 1999 to 2011 [13]. However, these studies were restricted to patients visiting an ED; and did not address important questions such as age and sex differences and trends over time; mortality differences and trends. Because there has been little recently-published work on asthma epidemiology in Alberta, we used population-based administrative health databases, and the same validated definition for asthma as OASIS, to assess province-wide asthma prevalence and incidence from 1995 to 2015, as well as mortality from 2000 to 2015. We present a more comprehensive picture of asthma in Alberta as we analyzed data from all physician–patient interactions with a diagnosis of asthma in the province and did not restrict our study to ED visits. We have noted differences in asthma incidence and prevalence between genders and age groups as well as over time. We also report the relative mortality of people with asthma compared to the general population. Tracking the trends over time can help inform decisions regarding interventions in the future.

## Methods

### Data sources

The study was approved by the Institutional Ethics Review board at the University of Alberta (Study ID: MS2_Pro00073225). The province of Alberta maintains a publicly funded, universally available, healthcare system for its population of 4.4 million residents. Coverage is available to all residents of the province. As part of managing the system, Alberta Health maintains several linkable population-based administrative databases.

De-identified data were provided by Alberta Health. The following databases were included: National Ambulatory Care Reporting System (NACRS), which records all ED visits and a number of non-emergency outpatient procedures; Discharge Abstract Database (DAD), which records all inpatient admissions and discharges; Physician Claims (Fee for Service), which records all visits to physicians and includes up to three ICD-9 diagnostic codes and inpatient physician billing codes (ICD-10); Alberta Health Care Insurance Program Central Stakeholder Registry, which holds demographic data for the population eligible for public health insurance; and, Vital Statistics Death Registration Database, which records cause of death.

### Study population

The study included all individuals residing in the province of Alberta, ages 0 to 99 from 1995 to 2015. The data collection algorithm used data starting from 1983 to be able to ensure accuracy of incidence and prevalence. As cases have been followed since 1983, the prevalence in 1995 was as a result of cases meeting the definition of asthma over the preceding 12 years, whereas incidence has been measured for each year starting in 1995. Individuals were included in the asthma cohort if they met the following criteria: two or more physician visits (outpatient or ED) for asthma within a 2-year period (visits must be on separate days) or one or more hospitalizations for asthma. This definition has been used in Ontario by the OASIS group (The Ontario Asthma Surveillance Information System) [7] and has been accepted as the asthma case definition by the Data Management Committee of the Alberta Respiratory Health Strategic Clinical Network [14]. Regarding the mortality data presented, we compared the general population cohort (those without asthma) and the asthma cohort from 2000 to 2015.

### Outcomes

The main outcomes were sex-specific, age-standardized prevalence and incidence of asthma from 1995 to 2015 and the trends over time. Age and sex-standardized mortality was assessed from 2000 to 2015, a period that used consistent physician billing codes (ICD-10) and was linkable to death information.

Prevalence data was defined as individuals in the cohort that have a claim or hospitalization for asthma within the study period. The date of first visit for asthma, or hospitalization for asthma was used to determine incidence. All-cause mortality was defined as yearly mortality in the asthma population from any cause, versus the all-cause yearly mortality in the general population, excluding individuals diagnosed with asthma.

### Statistical methods and analysis

Data was collected by the Alberta Health & Wellness Surveillance Branch using several algorithms. Records from 1983 were utilized in the lookback period to identify prevalent cases. The incidence and prevalence rates were standardized using the direct method and the 2005 population.

Data analysis was performed by the Alberta Strategy for Patient Oriented Research Support Unit (SPOR) branch of the Health Research Methods and Analytics, Faculty of Medicine and Dentistry, University of Alberta, and Alberta Health Services. The trends overtime were analyzed using Kendall’s Tau coefficient [15]. All analyses were completed using SAS 9.4 software (SAS Institute Inc, Cary, USA).

## Results

### Age-adjusted asthma prevalence in females and males

**Fig. 1:**
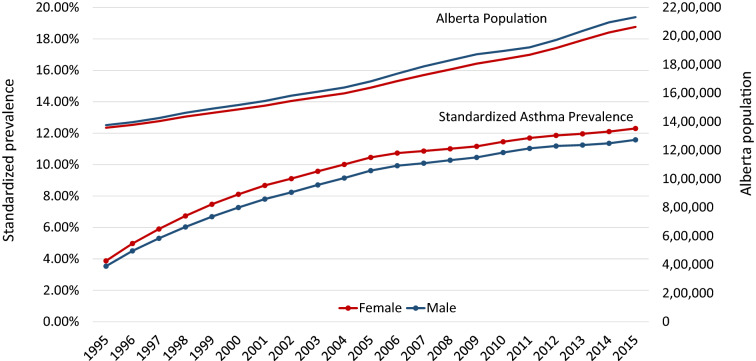
Age and sex adjusted asthma prevalence, from 1995 to 2015 compared to population growth

The age-adjusted prevalence of asthma tripled in both sexes over the duration of this study. In females the prevalence increased from 3.9% in 1995 to 12.3% in 2015, and in males from 3.5% in 1995 to 11.6% in 2015 (Fig. 1; Additional file 1: Table S1). Over time, the prevalence in females was consistently higher than in males. Over the same time-period, the total population in Alberta increased from ~ 2.7 to ~ 4.2 million (Fig. 1 and Additional file 1: Table S1 shows this increase for females and males separately). Thus, in 2015 there were 496,927 people with asthma in Alberta (248,943 females and 247,984 males). By 2015, 11.6% of the population of Alberta was living with a diagnosis of asthma.

The age-adjusted asthma prevalence was initially increasing dramatically from year to year; for example, there was a 28.5% increase in asthma prevalence in females and 27.6% increase in males from 1995 to 1996. However, from 2011 to 2015 the increase in prevalence was relatively stable at ~ 1–3% increase each year (Additional file 1: Table S2).

**Fig. 2:**
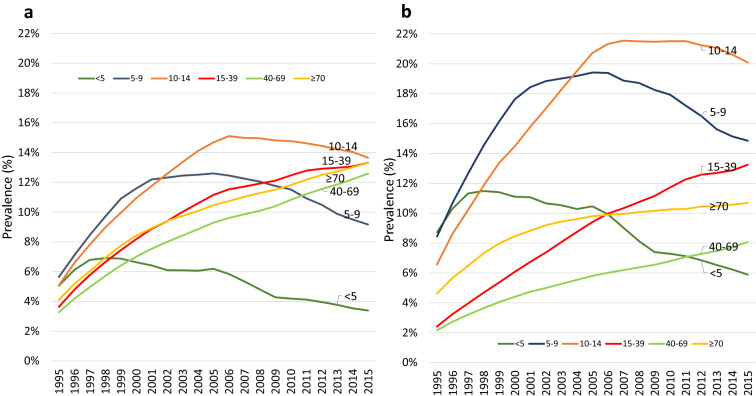
**a** Asthma prevalence in females and **b** asthma prevalence in males by age groups

The age distribution of asthma prevalence in females is shown in Fig. 2a, Additional file 1: Fig S1A and in Table S3. In females < 5 years old, the prevalence increased by 35% from 5.1% in 1995 to a peak of 6.9% in 1998 and was relatively stable until 2005. Thereafter it declined to 3.4% by 2015. In males < 5 years old, the prevalence increased by 30% from 8.7% in 1995 to a peak of 11.5% in 1998 and thereafter declined to 5.9% in 2015 (Fig. 2b). The prevalence in males was ~ 70% greater than in females age < 5 years old throughout the period of 1995–2015 (p < 0.0001) (Additional file 1: Table S3).

In the 5 to 9 year-old group, the prevalence in females more than doubled from 5.6% in 1995 to a peak of 12.6% in 2005 and thereafter decreased to 9.2% in 2015 (Fig. 2a; Additional file 1: Table S3). In males prevalence also more than doubled from 8.4% to a peak of 19.4% in 2005, and thereafter declined to 14.9% in 2015 (Fig. 2b; Additional file 1: Table S3). The prevalence in males of 5 to 9 years of age was greater than in females of the same age from 1995 to 2005. The difference between the sexes was up to 50% higher in males, with the largest difference in 2005 (p < 0.0001) (Additional file 1: Fig S1A and Table S3).

For children 10 to 14 years old, the prevalence in females tripled from 5.1% in 1995 to 15.1% in 2006 and thereafter was relatively stable, falling slightly to 13.7% by 2015 (Fig. 2a; Additional file 1: Table S3). In males the prevalence tripled from 6.6% in 1995 to 21.6% in 2007 and remained stable until 2015 (20.1%). The prevalence in males of 10 to 14 years of age was greater than in females throughout the study (p < 0.001) (Additional file 1: Fig S1a and Table S3).

For females in the age groups 15 to 39, 40 to 69 and ≥ 70 years, the trends of asthma prevalence more than tripled from 3.3–4.1% in 1995 to 12.6–13.3% in 2015 (Fig. 2a; Additional file 1: Table S3). The prevalence in males aged 15 to 39 also increased from 2.4% in 1995 to 13.2% in 2015. Similarly, in males aged 40 to 69 years, prevalence increased from 2.1 to 8.1%, and in males ≥ 70 years prevalence increased from 4.6 to 10.7% (Fig. 2b; Additional file 1: Table S3). Asthma prevalence in females was greater than in males in ages 15 to 39 and 40 to 69 from 1995 to 2015. In the ≥ 70 age group the prevalence was similar between the sexes until 2006 when the prevalence in females started increasing more than in the males until 2015 when the female prevalence was higher by 24%. The prevalence in these age groups was statistically higher in female population (p < 0.0001) (Additional file 1: Fig S1A and Table S3). Additional file 1: Table S3 shows that from 1995 to 2015, prevalence increased most dramatically in females 40–69 years old (285%) and males 15–39 years old (448%), whereas the < 5 age group of both genders had a decline in prevalence.

Additional file 1: Figure S1A highlights the difference in asthma prevalence between the sexes over time and across ages. The overall asthma prevalence was higher in females (p < 0.0001). Between 1995 and 2002 there were marked increases in prevalence in both females and males. While the peak prevalence in males occurred in the 5 to 9 age group in 2002, it changed to the 10 to 14 age group in 2009 and to the 15 to 19 age group in 2015. The prevalence in females was greater than in males after age 19 in 2002, after age 24 in 2009 and after age 29 in 2015.

### Age-adjusted asthma incidence in females and males

**Fig. 3:**
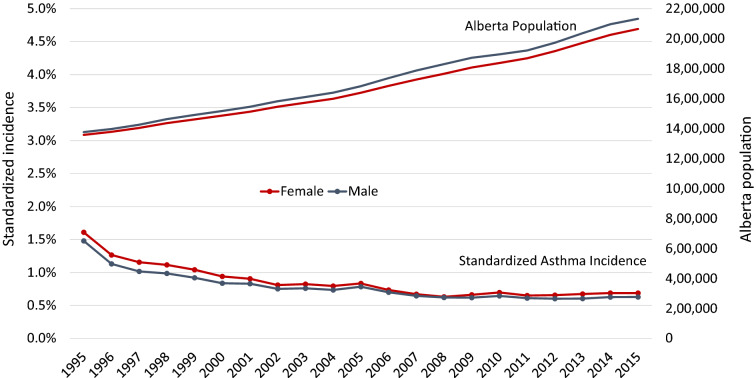
Age and sex adjusted asthma incidence from 1995 to 2015 compared to population growth

**Fig. 4:**
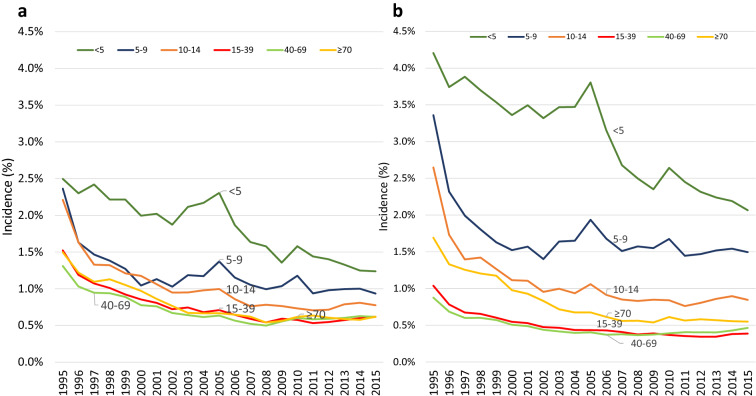
**a** Asthma incidence in females and **b** asthma incidence in males by age groups

The age-adjusted incidence of asthma in both females and males decreased with age. Incidence decreased over the 20 years by half from 1.5% in 1995 to 0.7% in 2015 (Fig. 3; Additional file 1: Tables S4 and S5). The incidence among all age groups in both females and males trended down between 1995 and 2015 and was most pronounced in the younger age groups of < 14 years (Fig. 4a, b, Additional file 1: Fig S1B; Table S6). Incidence in 2015 was higher for males under 14 than females. However, in the older age groups > 14, incidence was higher in females than males. The incidence was higher by 40% in males than females in those < 10 years old (< 5 years old and 5 to 9-year-old categories). In those 15–69 years old the incidence reversed and was higher in females by 25–40%. In ages 10–14 and ≥ 70 years old the incidence was similar between the sexes. From 2011 to 2015, incidence of asthma in both females and males had plateaued as seen in Fig. 3. The overall incidence was higher in females over the years (p < 0.0001). Of note, incidence in various age groups had changed over the years and comparing data from 1995, 2002, 2009 and 2015 (Additional file 1: Fig S1B), it is clear that while all incidence had decreased, the highest incidence was still in males < 14 years old. The second peak of higher incidence that was seen in 1995 for females and males > 70 years old was not present by 2015.

### Age-adjusted asthma prevalence and incidence in Alberta compared to Ontario

Using the OASIS data publicly available [16], we compared asthma prevalence and incidence in Alberta and Ontario from 1996 to 2015. We show that prevalence has been 25% higher in Ontario than in Alberta in females and males. In Alberta prevalence was 12.1% (females) and 11.6% (males), vs Ontario 15.9% (females) and 15.2% (males) (Table 1). However, over this time frame, incidence in females and males was higher in Alberta by ~ 15 to 40% compared to Ontario. Both provinces have had decreasing incidence at similar rates, decreasing overall by 45% in Alberta and by 60% in Ontario from 1996 to 2015. Similarly, prevalence increased in both provinces over the years, by 60% in Alberta and by 40% in Ontario in both males and females. Although the reports show different incidence and prevalence between the two provinces, the trends are concordant.

**Table 1:** Comparison of asthma prevalence and incidence between Alberta and Ontario from 1996 to 2015

Year	Alberta				Ontario			
	Prevalence		Incidence		Prevalence		Incidence	
	Female (%)	Male (%)	Female (%)	Male (%)	Female (%)	Male (%)	Female (%)	Male (%)
1996	5.04	4.77	1.29	1.20	9.23	8.34	0.98	0.89
2000	8.17	7.62	0.96	0.89	12.01	10.80	0.81	0.76
2005	10.49	9.96	0.87	0.86	13.92	12.71	0.60	0.60
2010	11.37	10.97	0.72	0.70	15.21	14.23	0.46	0.48
2015	12.06	11.63	0.70	0.67	15.94	15.16	0.37	0.38

### All-cause mortality in those with and without asthma

**Fig. 5:**
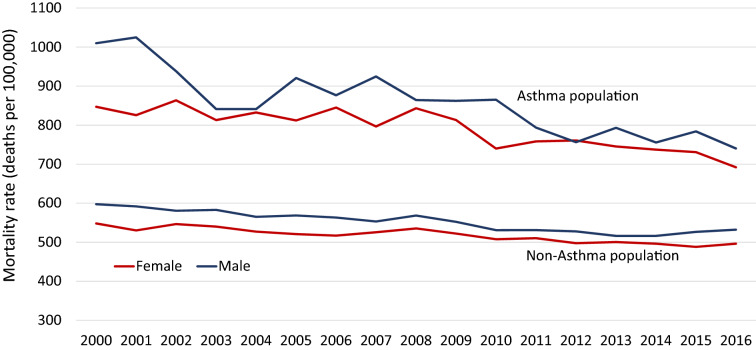
Comparison of all-cause mortality rates between the asthma and non-asthma population

Mortality in both the asthma and non-asthma populations declined over the 15 years of the study (mortality data starts from 2000). Age-adjusted all-cause mortality (deaths/100,000) in females and males with asthma was higher than in the non-asthma population of Alberta (p < 0.0001) in all years between 2000 and 2015 (Fig. 5; Additional file 1: Table S7). In 2015, the asthma population had a mortality rate 22% higher than the non-asthma population. The mortality rate in males was consistently higher than in females in both asthma and non-asthma populations.

Kendall’s tau coefficient is − 0.72 (p < 0.0001) in females and − 0.69 (p = 0.0001) in male, showing the strong decreasing trend of mortality rates in the asthma population over time. The decreasing trend in the non-asthma population is more obvious with tau = − 0.82 (p < 0.0001) in males and tau = − 0.74 (p < 0.0001) in females. Male mortality rates decreased faster than female rates in asthma population (p = 0.0001). Mortality rates in the non-asthma population decreased faster in males than in females (p < 0.0001), though slower than in the asthma population (Fig. 5).

Mortality decreased in groups over 40 years old in both the asthma and non-asthma populations, regardless of sex (males: Tau = − 0.6 to − 0.8, p < 0.01; females: Tau = − 0.5 to − 0.8, p < 0.01). For younger females with asthma, the mortality rate varied year by year without a significant trend (Tau = 0.3, p = 0.06 for ≤ 4 years; Tau = 0.2, p = 0.3 for 5–9 years; Tau = − 0.2, p = 0.3 for 10–14 years). This yearly variation was also observed in younger males (< 5 years old) with asthma, except that a decreasing trend was noted in the 5–9 years-old group (Tau = − 0.4, p = 0.02).

## Discussion

Survey-based epidemiologic studies of asthma from around the world have shown similar trends over time. To et al. (2012) published World Health Survey (WHS) data from 2002–2003 surveys across 70 countries showing an estimated asthma prevalence of 4.5% of adults (18–45 years old) with wide variation depending on geographic area [1]. The CDC reported in 2015 that 7.6% of people over 18 years of age, 18.4 million, and 8.4% of children had a diagnosis of asthma, making it 8.2% of the entire US population [2]. The prevalence of asthma in the US increased from 2001 to 2010 to 8.4%, and then stabilized at 8.2% in 2015 [2, 3]. Australia saw similar trends of increases in the prevalence of asthma in children between 1982 and 1992, with decreases or stabilization from 1992 to 2002 [17]. A Statistics Canada Health Survey showed that in 2014, 8.1% of Canadians aged 12 and older, 2.4 million people, had been diagnosed with asthma by a health professional in the community. Using telephone surveys of a sample of the population, Statistics Canada estimates that 274,661 people were living with asthma in Alberta in 2014, which translates to approximately 8.1% of the population that was 12 years old and older at that time [4]. Using health databases, we have identified that amongst Albertans of all ages, there were 240,777 (11.9%) females and 239,560 (11.4%) males living with asthma in 2014 (Additional file 1: Table S1).

Although there are no recent publications on the prevalence of asthma in Alberta, there are several publications reporting on emergency care for asthma in Alberta that make use of databases. A study on the rates of asthma presentations in ED from 1999 to 2011 showed that the number of ED presentations has been declining during that period [10]. Rosychuk 2010 showed that during a similar period (1999–2005) there were higher rates of ED visits in non-urban areas of Alberta compared to Edmonton and Calgary, with crude rates in 2004/2005 of 7.9/1000, 6.5/1000 and 15.4/1000 in the Edmonton, Calgary and non-urban areas, respectively. These trends were stable over the study period [11]. Smaller and older, survey-based studies tended to focus on specific populations. The prevalence of asthma in 2001 in children aged 5 to 19 years old in two cities in Alberta, Red Deer and Medicine Hat, showed the prevalence of asthma was higher in Medicine Hat (17.0%) than in Red Deer (12.8%) [18]. These estimates were based on surveys sent out to students at several schools in each city and were followed up by telephone surveys.

A great example of the use of databases to inform more accurate estimates of incidence and prevalence is seen in Ontario, where OASIS has reported on asthma epidemiology since 2003, with a variety of topics. Specifically, in 2010 Gershon et al*.* showed that from 1996 to 2005 the asthma prevalence in Ontario increased from 8.5% in 1996 to 13.3% in 2005. Asthma incidence rates increased in children by 30.0% and were relatively stable in adults as all-cause mortality decreased. Asthma prevalence in Ontario, had increased partly due to the increase in childhood asthma incidence [9]. The same group calculated the lifetime risk of physician-diagnosed asthma was 33.9% in Ontario in 2010 [19] and forecasted that in 2022 there will be more than 71,000 new cases of asthma and more than 1.9 million individuals living with asthma [20]. More recently, the group reported the asthma prevalence in 2016 as 15.5% and incidence 0.25%. We show that Alberta has (Table 1, Additional file 1: Tables S2 and S4) similar trends as Ontario, with decreasing incidence, but a steady increase in prevalence. Fewer patients were being diagnosed with asthma yearly, but the prevalence continued to steadily increase, although at a slower rate. Our data closely resembles that presented by OASIS. We have shown that in Alberta, while asthma incidence has decreased from 1.5% in 1995 to 0.7% in 2015, prevalence increased by 0.25% to 2% each year. In comparing mortality in males and females diagnosed with asthma, we furthermore show a decrease in overall mortality. Despite the decrease in overall population mortality, there is still higher all-cause mortality in those diagnosed with asthma. These differences in mortality are also evident in the Ontario data. In 2008 in Ontario there were 639.9/100,000 deaths from all causes in the general (non-asthma) population compared to 852.1/100,000 in those diagnosed with asthma [25]. Comparatively, in Alberta, in 2009, there were 522.1/100,000 deaths in the non-asthma population versus 813.1/100,000 deaths in the asthma population. We have used similar methods and the same definition of asthma, therefore, comparison with the Ontario data provides important information on asthma in two separate jurisdictions in Canada.

Several studies around the world have estimated mortality rates in patients with asthma and have shown that mortality directly due to asthma is not reported frequently, but the risk of death from any cause in patients with asthma is higher than in those without. A recent European study used data from Netherlands, Italy, UK, Denmark and Spain from 2008 to 2013, and evaluated 586,436 adult asthma patients, estimating that the age and sex standardized all-cause mortality rate was 5.2 to 9.5/1000 person-years in asthma. Mortality rates were higher in the first month following a severe asthma exacerbation and decreased thereafter; thus, showing that asthma is an important factor leading to increased mortality [21]. Similarly, 54,320 adult subjects were examined in seven independent cross-sectional population surveys repeated every 5 years between 1982 and 2012 in Finland. All-cause mortality decreased between 1982 and 2015, though mortality in asthmatic subjects compared with non-asthmatics was higher from all causes [22]. Another study from Finland looked at a smaller number of patients over the age of 30. This was a combination of surveys and database use. This study is important as it had a control non-asthma group. Over 15.6 years, there were 221 deaths among 1052 asthma patients and 335 deaths among 1889 non-asthma patients. Asthma was associated with increased all-cause mortality (adjusted HR 1.25; 95% CI 1.05–1.49, p = 0.011) [23].

Rates of death from asthma in the United States increased from 0.8/100,000 in 1977 to 2.1 in 1994 but decreased to 1.6/100,000 in 2000. Rates had been higher for women than men but have overall decreased for both [24]. The CDC published that asthma deaths further decreased from 15 per million in 2001 to 11 per million in 2015. Adults were more likely than children to die from asthma. The asthma death rate was highest among the 65 years and older age group compared with all other age groups [2]. As mentioned above, in Ontario in 2008, the asthma population had higher all-cause mortality compared with the general population (rate ratio, 1.3) and total mortality in those with asthma was fourfold higher than mortality directly due to asthma [25]. All-cause mortality rates in asthma patients have decreased substantially over the past 20 years as data from Ontario and now Alberta have shown. Compared with the general population however, the asthma population has a higher all-cause mortality and is more likely to die from comorbid conditions. In Alberta, the asthma population had a mortality rate 22% higher than the non-asthma population in 2015, adding to the increasing evidence that an asthma diagnosis is associated with higher mortality.

### Strengths and limitations of our study

As research in the field progresses, multiple asthma phenotypes have been identified which add to the difficulty of the diagnosis. Because it is a heterogeneous, chronic disease and requires long-term follow-up for diagnosis, it is also difficult to study its epidemiology. Socioeconomic factors that may impair access to healthcare, the association and overlap with COPD, multiple phenotypes and difficulty in predicting disease courses are some of the barriers to the accurate diagnosis of asthma. Diagnostic code usage could vary among practitioners. An asthma presentation may be coded as bronchitis, dyspnea, respiratory distress, etc. Objective assessment with pulmonary function testing and spirometry are not used for every patient that is diagnosed with asthma as noted by the Ontario group and by the Respiratory Health—Strategic Clinical Network of Alberta (authors of this study are member of the reporting group) [26]. Physicians have identified difficulty in continuity of care as a problem in the diagnosis of asthma. Less than half of the patients with new physician-diagnosed asthma in Ontario received objective pulmonary function testing around the time of diagnosis [27].

Given the heterogeneity in epidemiological studies regarding definitions, study methods, geographic areas, age groups and population subsections studied, it is not surprising that there have been conflicting reports of the incidence and prevalence of asthma [28]. Accuracy begins with the clinical asthma diagnosis, but according to a recent publication as many as 33% of asthma patients may be misdiagnosed as having asthma [29]. Misdiagnosis is likely multifactorial and includes lack of pulmonary function testing, as well as difficulty establishing continuity of care [26, 27]. Furthermore, the definition of asthma in population studies has varied widely from survey questions of a prior diagnosis, current or past symptoms, one or several ED visits, and multiple health claims for asthma [30]. This leads to heterogeneous data that cannot be compared or used to estimate changes in incidence and prevalence over time.

Brogger et al*.* in 2004 showed that survey-based retrospective estimates of trends in asthma incidence are likely to be severely biased by differential recall [5]. In recent years, there has been increasing use of database research to improve the accuracy of estimates and to follow trends over time in a more efficacious and reliable way. For database type studies, OASIS has shown that the algorithm of two or more ambulatory care visits and/or one or more hospitalization(s) for asthma in 2 years allows for accurate statistical investigations in both adults and children [7, 8]. A large, single, publicly funded healthcare system such as Alberta Health, facilitates database research. This improves the accuracy of the data we present over studies based on survey methods or using smaller populations.

Sources of error in the data collection include different access to healthcare for various groups including lower socio-economic, First Nations, remote geographic areas, and different age groups. There are several other potential sources of error in our estimates. The data was collected starting in 1983 and if a patient with asthma had not met that diagnosis within the past 2 years then they were not included in the prevalence and may either become part of the incidence and then prevalence or may never be picked up. With a long period of latency, certain cases of childhood asthma that become symptomatic in adulthood again, may be difficult to ascertain by the duration of the study. In our study, the accuracy increased by a long look-back period that started in 1983. Other challenges include immigration and emigration as people with asthma may come into the province and leave, leading to cases that are missed. It is very likely that there are also cases of overdiagnosis, especially in the pediatric population. Starting in the early to mid-1990′s there was an increase in asthma awareness which may have influenced the increase in diagnosis and therefore incidence [31]. Indeed, recommendations of the Canadian Asthma Consensus Report of 1999 may have also impacted physician awareness and diagnosis rates [32]. The 2003 Canadian Asthma Consensus Guidelines Executive Summary highlighted the importance of early diagnosis of asthma and included criteria supporting a diagnosis of asthma in preschool children [33].

### Practical applications of epidemiologic studies

There is need for a unified, accurate system for epidemiological research that can improve resource allocation and therefore clinical care and outcomes. If we can harness data from all provinces and territories and more accurately describe the epidemiology of asthma across the country, we will be better able to fill in gaps in health care resource allocation. While the goal of this study was to provide the essential statistics regarding incidence, prevalence, and mortality, we hope this is a starting point for future studies that will address further issues related to asthma in Alberta in a more detailed fashion. We wish to highlight that more extensive studies using databases could provide further information regarding medication use, pulmonary function testing, asthma severity, comorbidities, geographic differences (including urban versus rural differences), and cost to the healthcare system.

Having accurate measurements of the state of a disease fosters the allocation of resources in a responsible manner. Over the past several years, Alberta’s annual health care budgets have been over 20 billion dollars, about 43% of the total annual budget. Strategizing healthcare funding and investments can lead to more efficient and effective overall spending. For example, an accurate asthma diagnosis with pulmonary function testing and then proper treatment with general practitioner follow-up can lead to less ED visits, hospitalizations, time off-work, impaired lung function and mortality which could translate into cost savings in the long term. Descriptive epidemiological studies that can accurately identify the burden of chronic disease such as asthma are important for disease monitoring over time, resource allocation and measuring the effectiveness of interventions [34].

## Conclusions

We have shown that the incidence of asthma decreased in both females and males over 20 years in Alberta. While prevalence continued to increase over the years, it did so at a slower rate. Our data has demonstrated similar trends to the incidence and prevalence of asthma in Ontario. The data comparing incidence between age groups and sexes demonstrated that in 2015 asthma was diagnosed more often in males ages 0 to 29 and in females aged 30 and older. All-cause mortality in asthma patients was shown to be higher than in those without asthma. This difference between the two populations was not affected by the steady overall decline in mortality. We conclude that an asthma diagnosis is associated with increased risk of overall mortality.

## Supplementary information

**Additional file 1: Table S1.** Standardized Asthma prevalence male vs. female.** Table S2**. The rate of change in prevalence from year to year.** Figure S1.** A. Asthma prevalence and B. Asthma incidence in females and males stratified by age.** Table S3.** Age-adjusted asthma prevalence in males vs females and in different age groups in 1995, 2002, 2009, 2015.** Table S4.** The rate of change in incidence from one year to the next.** Table S5.** Standardized Asthma incidence male vs. female.** Table S6.** Age-adjusted asthma incidence in males vs females and in different age groups in 1995, 2002, 2009, 2015.** Table S7. **Age and sex specific all-cause mortality rates*(number of deaths per 100,000) of populations with and without asthma in Alberta in 2000, 2002, 2009, 2015.

## Data Availability

The data that support the findings of this study are available from Alberta Health but restrictions apply to the availability of these data, which were used under license for the current study, and so are not publicly available. Data are however available from the authors upon reasonable request and with permission of Alberta Health.

## Abbreviations

OASIS: Ontario Asthma Surveillance Information System
ED: Emergency department
NACRS: National Ambulatory Care Reporting System
DAD: Discharge Abstract Database
SPOR: Strategy for Patient Oriented Research Support Unit
ICD-9: International Classification of Diseases, Ninth Revision
ICD-10: International Classification of Diseases, Tenth Revision
WHS: World Health Survey

## References

[1] Global Asthma Network. The Global Asthma Report 2018. Auckland, New Zealand. 2018. 92 p. www.globalasthmanetwork.org. Accessed Sept 2019.

[2] Centers for Disease Control [CDC]. National Current Asthma Prevalence (2015). National Health Interview Survey (NHIS) Data. 2015. Accessed Sept 2019.

[3] Moorman JE, Akinbami LJ, Bailey CM, Zahran HS, King ME, Johnson CA, et al. National surveillance of asthma: United States, 2001–2010. Vital Health Stat 3. 2012. 24252609

[4] Statistics Canada. Canadian Community Health Survey, 2014: annual component. Cycle 1.1. 2014. Accessed Sept 2019.

[5] Brogger J, Eagan T, Eide GE, Bakke P, Gulsvik A (2004). Bias in retrospective studies on trends in asthma incidence. Eur Respir J.

[6] Huzel L, Roos LL, Anthonisen NR, Manfreda J (2002). Diagnosing asthma: the fit between survey and administrative database. Can Respir J.

[7] Gershon AS, Wang C, Guan J, Vasilevska-Ristovska J, Cicutto L, To T (2009). Identifying patients with physician-diagnosed asthma in health administrative databases. Can Respir J.

[8] To T, Dell S, Dick PT, Cicutto L, Harris JK, MacLusky IB (2006). Case verification of children with asthma in Ontario. Pediatr Allergy Immunol.

[9] Gershon AS, Guan J, Wang C, To T (2010). Trends in asthma prevalence and incidence in Ontario, Canada, 1996–2005: a population study. Am J Epidemiol.

[10] Rosychuk RJ, Voaklander DC, Klassen TR, Senthilselvan A, Marrie TJ, Rowe BH (2010). A population-based study of emergency department presentations for asthma in regions of Alberta. Can J Emerg Med.

[11] Rosychuk RJ, Voaklander DC, Klassen TP, Senthilselvan A, Marrie TJ, Rowe BH (2010). Asthma presentations by children to emergency departments in a Canadian province: a population-based study. Pediatr Pulmonol.

[12] Rowe BH, Voaklander DC, Wang D, Senthilselvan A, Klassen TP, Marrie TJ (2009). Asthma presentations by adults to emergency departments in Alberta, Canada: a large population-based study. Chest.

[13] Rosychuk RJ, Youngson E, Rowe BH (2015). Presentations to Alberta emergency departments for asthma: a time series analysis. Acad Emerg Med.

[14] Sharpe H, Fong A, Kabir S, To TM, Gershon AS, Respiratory Health Strategic Clinic MWG, et al. Asthma and COPD prevalence, incidence and mortality in Alberta and Ontario, Canada. In: American Thoracic Society Meeting, Dallas TX, A3017. 2019.

[15] Kendall MG (1938). A new measure of rank correlation. Biometrika.

[16] OASIS. Ontario asthma surveillance. https://lab.research.sickkids.ca/oasis/data-tables/. Accessed Sept 2019.

[17] Toelle BG, Ng K, Belousova E, Salome CM, Peat JK, Marks GB (2004). Prevalence of asthma and allergy in schoolchildren in Belmont, Australia: three cross sectional surveys over 20 years. Br Med J.

[18] Hessel PA, Klaver J, Michaelchuk D, McGhan S, Carson MM, Melvin D (2001). The epidemiology of childhood asthma in Red Deer and Medicine Hat, Alberta. Can Respir J.

[19] To T, Wang C, Guan J, McLimont S, Gershon AS (2010). What is the lifetime risk of physician-diagnosed asthma in Ontario, Canada?. Am J Respir Crit Care Med.

[20] To T, Stanojevic S, Feldman R, Moineddin R, Atenafu EG, Guan J (2013). Is asthma a vanishing disease? A study to forecast the burden of asthma in 2022. BMC Public Health.

[21] Engelkes M, de Ridder MA, Svensson E, Berencsi K, Prieto-Alhambra D, Lapi F (2020). Multinational cohort study of mortality in patients with asthma and severe asthma. Respir Med.

[22] Pelkonen MK, Notkola ILK, Laatikainen TK, Jousilahti P (2018). 30-year trends in asthma and the trends in relation to hospitalization and mortality. Respir Med.

[23] Lemmetyinen RE, Karjalainen JV, But A, Renkonen RLO, Pekkanen JR, Toppila-Salmi SK (2018). Higher mortality of adults with asthma: a 15-year follow-up of a population-based cohort. Allergy Eur J Allergy Clin Immunol.

[24] Sly RM (2004). Continuing decreases in asthma mortality in the United States. Ann Allergy Asthma Immunol.

[25] To T, Simatovic J, Zhu J, Feldman L, Dell SD, Lougheed MD (2014). Asthma deaths in a large provincial health system: a 10-year population-based study. Ann Am Thorac Soc.

[26] Sharpe H, Claveria-Gonzalez FC, Davidson W, Befus AD, Leung JP, Young E (2020). Adulta asthma diagnosis: physician reported challenges in Alberta-based primary care practices. SAGE Open Nurs.

[27] Gershon AS, Victor JC, Guan J, Aaron SD, To T (2012). Pulmonary function testing in the diagnosis of asthma: a population study. Chest.

[28] Lawson JA, Senthilselvan A (2005). Asthma epidemiology: has the crisis passed?. Curr Opin Pulm Med.

[29] Aaron SD, Vandemheen KL, FitzGerald JM, Ainslie M, Gupta S, Lemière C (2017). Reevaluation of diagnosis in adults with physician-diagnosed asthma. JAMA.

[30] Yiannakoulias N, Schopflocher DP, Svenson LW (2009). Using administrative data to understand the geography of case ascertainment. Chronic Dis Can.

[31] Hargreave FE, Dolovich J, Newhouse MT (1990). The assessment and treatment of asthma: a conference report. J Allergy Clin Immunol.

[32] Boulet LP, Becker A, Berube D, Beveridge R, Ernst P, Acres JC, et al. Summary of recommendations from the Canadian Asthma consensus report, 1999. In: CMAJ. 1999. p. 161 (11 suppl 2) S1–S12. PMC1230847 10906907

[33] Becker A, Lemière C, Bérubé D, Boulet L-P, Ducharme F, FitzGerald M (2006). 2003 Canadian asthma consensus guidelines executive summary. Allergy Asthma Clin Immunol.

[34] To T, Guan J, Zhu J, Lougheed MD, Kaplan A, Tamari I (2015). Quality of asthma care under different primary care models in Canada: a population-based study. BMC Fam Pract.

