# Supplementary material for: Developments in asthma incidence and prevalence in Alberta between 1995 and 2015
Source: Allergy Asthma Clin Immunol. 2020 Oct 9;16:87. doi: 10.1186/s13223-020-00485-3 (PMC7547457; doi:10.1186/s13223-020-00485-3)
Supplement: Supplementary file 1 — Additional file 1: Table S1. Standardized Asthma prevalence male vs. female. Table S2. The rate of change in prevalence from year to year. Figure S1. A. Asthma prevalence and B. Asthma incidence in females and males stratified by age. Table S3. Age-adjusted asthma prevalence in males vs females and in different age groups in 1995, 2002, 2009, 2015. Table S4. The rate of change in incidence from one year to the next. Table S5. Standardized Asthma incidence male vs. female. Table S6. Age-adjusted asthma incidence in males vs females and in different age groups in 1995, 2002, 2009, 2015. Table S7. Age and sex specific all-cause mortality rates*(number of deaths per 100,000) of populations with and without asthma in Alberta in 2000, 2002, 2009, 2015. [file 13223_2020_485_MOESM1_ESM.pptx]

## Slide 1
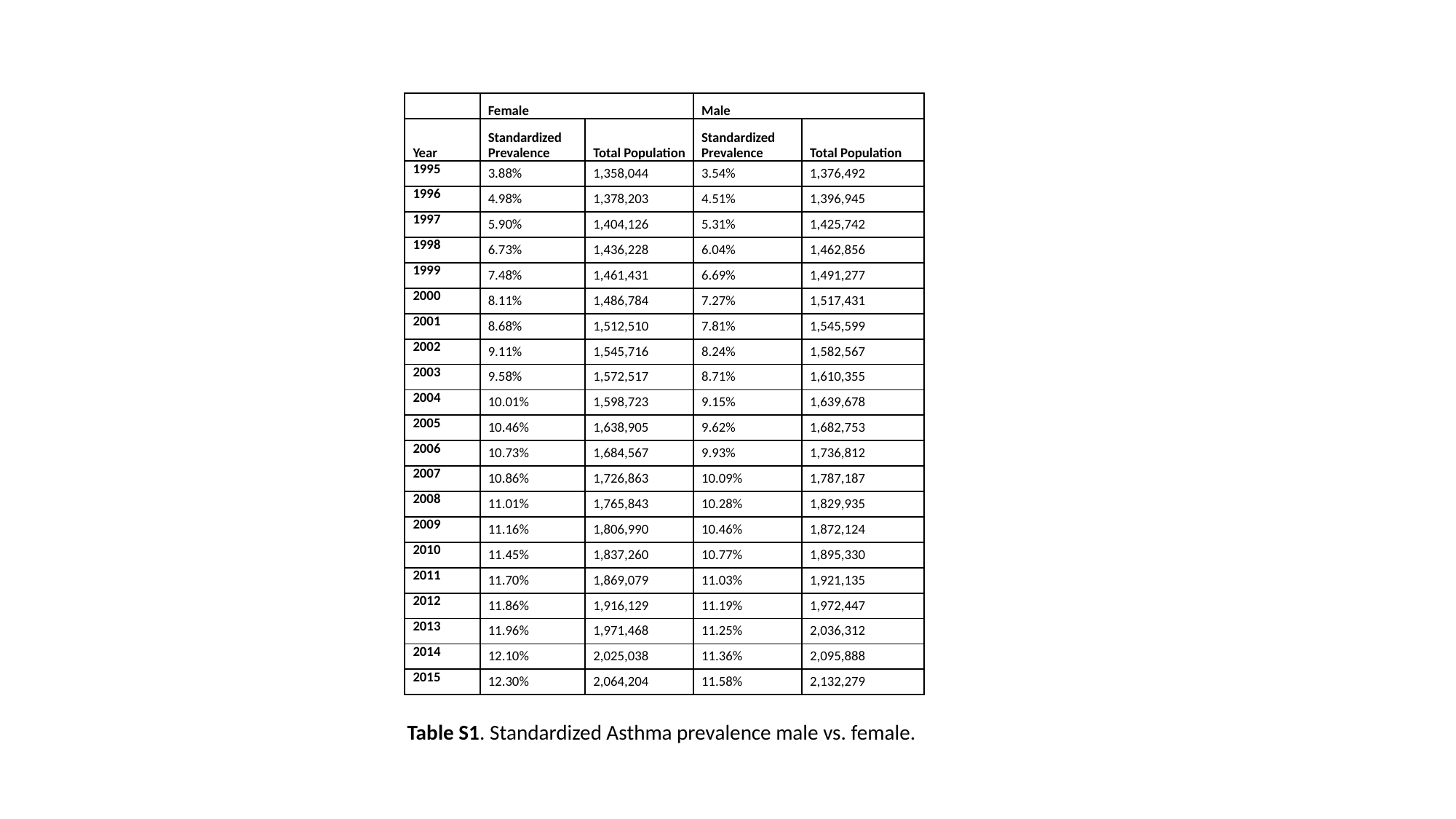

| | Female | | Male | |
| --- | --- | --- | --- | --- |
| Year | Standardized Prevalence | Total Population | Standardized Prevalence | Total Population |
| 1995 | 3.88% | 1,358,044 | 3.54% | 1,376,492 |
| 1996 | 4.98% | 1,378,203 | 4.51% | 1,396,945 |
| 1997 | 5.90% | 1,404,126 | 5.31% | 1,425,742 |
| 1998 | 6.73% | 1,436,228 | 6.04% | 1,462,856 |
| 1999 | 7.48% | 1,461,431 | 6.69% | 1,491,277 |
| 2000 | 8.11% | 1,486,784 | 7.27% | 1,517,431 |
| 2001 | 8.68% | 1,512,510 | 7.81% | 1,545,599 |
| 2002 | 9.11% | 1,545,716 | 8.24% | 1,582,567 |
| 2003 | 9.58% | 1,572,517 | 8.71% | 1,610,355 |
| 2004 | 10.01% | 1,598,723 | 9.15% | 1,639,678 |
| 2005 | 10.46% | 1,638,905 | 9.62% | 1,682,753 |
| 2006 | 10.73% | 1,684,567 | 9.93% | 1,736,812 |
| 2007 | 10.86% | 1,726,863 | 10.09% | 1,787,187 |
| 2008 | 11.01% | 1,765,843 | 10.28% | 1,829,935 |
| 2009 | 11.16% | 1,806,990 | 10.46% | 1,872,124 |
| 2010 | 11.45% | 1,837,260 | 10.77% | 1,895,330 |
| 2011 | 11.70% | 1,869,079 | 11.03% | 1,921,135 |
| 2012 | 11.86% | 1,916,129 | 11.19% | 1,972,447 |
| 2013 | 11.96% | 1,971,468 | 11.25% | 2,036,312 |
| 2014 | 12.10% | 2,025,038 | 11.36% | 2,095,888 |
| 2015 | 12.30% | 2,064,204 | 11.58% | 2,132,279 |
Table S1. Standardized Asthma prevalence male vs. female.

## Slide 2
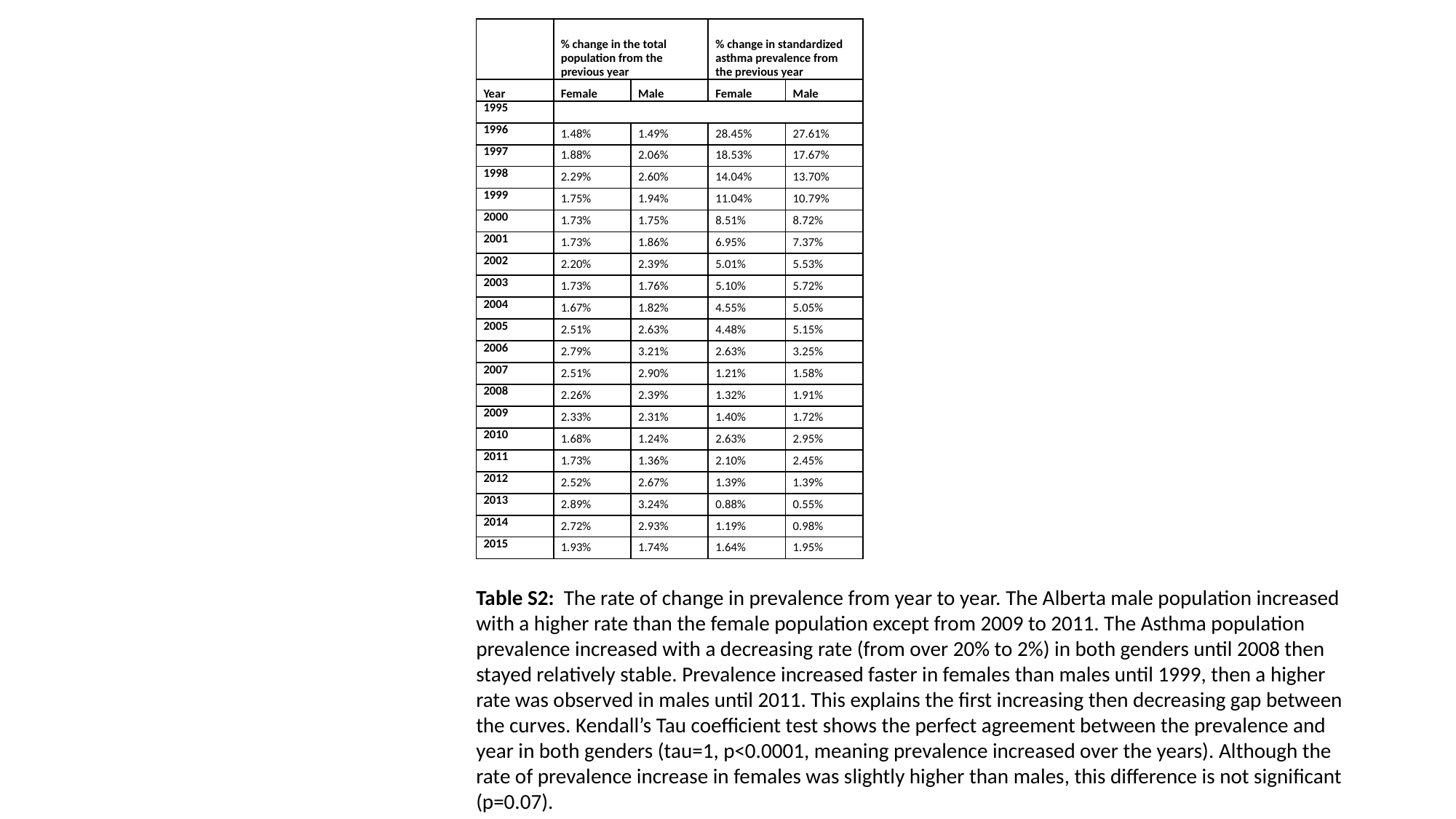

| | % change in the total population from the previous year | | % change in standardized asthma prevalence from the previous year | |
| --- | --- | --- | --- | --- |
| Year | Female | Male | Female | Male |
| 1995 | | | | |
| 1996 | 1.48% | 1.49% | 28.45% | 27.61% |
| 1997 | 1.88% | 2.06% | 18.53% | 17.67% |
| 1998 | 2.29% | 2.60% | 14.04% | 13.70% |
| 1999 | 1.75% | 1.94% | 11.04% | 10.79% |
| 2000 | 1.73% | 1.75% | 8.51% | 8.72% |
| 2001 | 1.73% | 1.86% | 6.95% | 7.37% |
| 2002 | 2.20% | 2.39% | 5.01% | 5.53% |
| 2003 | 1.73% | 1.76% | 5.10% | 5.72% |
| 2004 | 1.67% | 1.82% | 4.55% | 5.05% |
| 2005 | 2.51% | 2.63% | 4.48% | 5.15% |
| 2006 | 2.79% | 3.21% | 2.63% | 3.25% |
| 2007 | 2.51% | 2.90% | 1.21% | 1.58% |
| 2008 | 2.26% | 2.39% | 1.32% | 1.91% |
| 2009 | 2.33% | 2.31% | 1.40% | 1.72% |
| 2010 | 1.68% | 1.24% | 2.63% | 2.95% |
| 2011 | 1.73% | 1.36% | 2.10% | 2.45% |
| 2012 | 2.52% | 2.67% | 1.39% | 1.39% |
| 2013 | 2.89% | 3.24% | 0.88% | 0.55% |
| 2014 | 2.72% | 2.93% | 1.19% | 0.98% |
| 2015 | 1.93% | 1.74% | 1.64% | 1.95% |
Table S2: The rate of change in prevalence from year to year. The Alberta male population increased with a higher rate than the female population except from 2009 to 2011. The Asthma population prevalence increased with a decreasing rate (from over 20% to 2%) in both genders until 2008 then stayed relatively stable. Prevalence increased faster in females than males until 1999, then a higher rate was observed in males until 2011. This explains the first increasing then decreasing gap between the curves. Kendall’s Tau coefficient test shows the perfect agreement between the prevalence and year in both genders (tau=1, p<0.0001, meaning prevalence increased over the years). Although the rate of prevalence increase in females was slightly higher than males, this difference is not significant (p=0.07).

## Slide 3
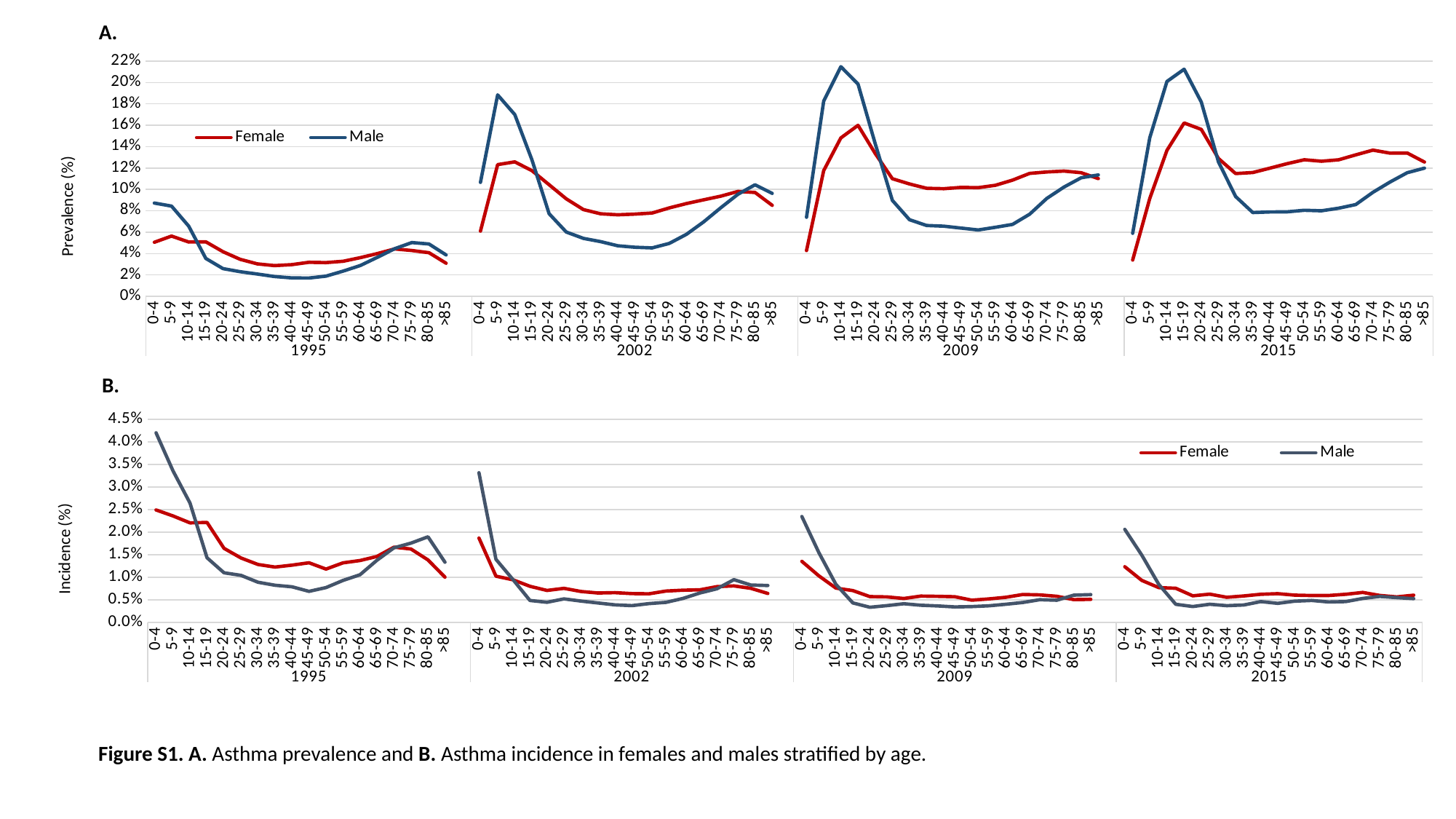

A.
### Chart
| Category | Female | Male |
|---|---|---|
| 0-4 | 0.05056759545923633 | 0.08728102995068977 |
| 5-9 | 0.05637311480455525 | 0.08443546691771243 |
| 10-14 | 0.05082533513906063 | 0.06567366912900284 |
| 15-19 | 0.0509991339769702 | 0.035445973881913985 |
| 20-24 | 0.04175594515574039 | 0.025935554077746205 |
| 25-29 | 0.034622570641601014 | 0.023051745199884368 |
| 30-34 | 0.030361357983619282 | 0.02088644984764629 |
| 35-39 | 0.028747400795376382 | 0.018540782917073097 |
| 40-44 | 0.029633993528960706 | 0.01727740776638597 |
| 45-49 | 0.031838929814254076 | 0.017160720531654426 |
| 50-54 | 0.031561436207082454 | 0.018923288819689802 |
| 55-59 | 0.03283001601464196 | 0.023605947955390335 |
| 60-64 | 0.03618860674908119 | 0.02882003308891113 |
| 65-69 | 0.040033895975997984 | 0.03655352480417755 |
| 70-74 | 0.04433831780578825 | 0.044542092118989664 |
| 75-79 | 0.0428800893917173 | 0.05028281085226728 |
| 80-85 | 0.040797151617050734 | 0.0489763779527559 |
| >85 | 0.03095883948578842 | 0.03870967741935484 |
| | None | None |
| 0-4 | 0.06100050228164107 | 0.10659277972455383 |
| 5-9 | 0.12320467446397584 | 0.18840593107528472 |
| 10-14 | 0.12577205849106085 | 0.16998760524853615 |
| 15-19 | 0.11754049445865303 | 0.12758932497117212 |
| 20-24 | 0.10440851541402167 | 0.07731385676607831 |
| 25-29 | 0.09121600555847534 | 0.060158276688667786 |
| 30-34 | 0.08116023854052719 | 0.05418447878987175 |
| 35-39 | 0.07722835398653226 | 0.05120180748181451 |
| 40-44 | 0.07629227213167852 | 0.0473028458162254 |
| 45-49 | 0.07695529693903531 | 0.04592860797355268 |
| 50-54 | 0.07793332371193534 | 0.04529972752043597 |
| 55-59 | 0.08274009402283412 | 0.04948044212059038 |
| 60-64 | 0.08679367013745766 | 0.05798328409827797 |
| 65-69 | 0.09030079419673566 | 0.06959414216903903 |
| 70-74 | 0.09370093285305256 | 0.08285368070281733 |
| 75-79 | 0.09814130556488634 | 0.0954661673088696 |
| 80-85 | 0.09715470133785566 | 0.10433405665356871 |
| >85 | 0.08513184324116416 | 0.09632034632034632 |
| | None | None |
| 0-4 | 0.042784827681852856 | 0.07397811845079952 |
| 5-9 | 0.11762031878675333 | 0.18254972463702152 |
| 10-14 | 0.14816066649005655 | 0.2147622659724856 |
| 15-19 | 0.1599531116794544 | 0.19846886509903894 |
| 20-24 | 0.13363525215768265 | 0.14283588248425524 |
| 25-29 | 0.11002193217074997 | 0.08977668535856327 |
| 30-34 | 0.1051015500721394 | 0.07162511919357036 |
| 35-39 | 0.10106506135679555 | 0.0662379375624836 |
| 40-44 | 0.10069264002109721 | 0.06559866427733517 |
| 45-49 | 0.10189553787616741 | 0.0638611504268933 |
| 50-54 | 0.1016681644150097 | 0.06207047247147056 |
| 55-59 | 0.10387260224393775 | 0.06456655699561922 |
| 60-64 | 0.1087053598368273 | 0.06727054589082183 |
| 65-69 | 0.11500656410946915 | 0.07684916669578282 |
| 70-74 | 0.11634025759086762 | 0.0916104374085783 |
| 75-79 | 0.11716102219033718 | 0.10222286793549325 |
| 80-85 | 0.11565796602607005 | 0.11091954022988505 |
| >85 | 0.11015447735678985 | 0.11355073405222814 |
| | None | None |
| 0-4 | 0.033926981300089046 | 0.058927044389633705 |
| 5-9 | 0.09167242136982862 | 0.14848864432812897 |
| 10-14 | 0.13656232374506486 | 0.20094821603271096 |
| 15-19 | 0.16216535965386694 | 0.21236717124386487 |
| 20-24 | 0.1561293795193949 | 0.1817238073197342 |
| 25-29 | 0.1289180871212121 | 0.1256879285456514 |
| 30-34 | 0.1148443027029455 | 0.09337272300669766 |
| 35-39 | 0.1158057172736142 | 0.07840973825442989 |
| 40-44 | 0.11988711488974509 | 0.07893747650670342 |
| 45-49 | 0.1240813806397205 | 0.07905731269627636 |
| 50-54 | 0.1277269406074945 | 0.08053185336340064 |
| 55-59 | 0.12638849857267523 | 0.08003090695197589 |
| 60-64 | 0.12768510630501892 | 0.08246554703770993 |
| 65-69 | 0.13233347003339388 | 0.08587085272616327 |
| 70-74 | 0.13674909866450008 | 0.09733092144122858 |
| 75-79 | 0.13393136772403919 | 0.10695501268188493 |
| 80-85 | 0.13403991820512082 | 0.11564702354176039 |
| >85 | 0.125632823716092 | 0.11989878076834598 |B.
### Chart
| Category | Female | Male |
|---|---|---|
| 0-4 | 0.024957659258335672 | 0.04204760562245761 |
| 5-9 | 0.02362635722176332 | 0.03359285307709377 |
| 10-14 | 0.022092353113656815 | 0.02647395257759941 |
| 15-19 | 0.02218672541999449 | 0.01435431350727505 |
| 20-24 | 0.016445835996663164 | 0.011028132992327366 |
| 25-29 | 0.014307628768658917 | 0.010456022366631405 |
| 30-34 | 0.012870137410489646 | 0.008930187179870493 |
| 35-39 | 0.012293467336683418 | 0.008267241247574077 |
| 40-44 | 0.01272037066988878 | 0.007926462535570337 |
| 45-49 | 0.013242745278673423 | 0.0069136893257496276 |
| 50-54 | 0.011850315117795903 | 0.00775252487016035 |
| 55-59 | 0.01322725592795036 | 0.009335395292697647 |
| 60-64 | 0.013740197021176572 | 0.010591809555518562 |
| 65-69 | 0.014645728525083454 | 0.013804094642638225 |
| 70-74 | 0.016701573492293648 | 0.01656200175706895 |
| 75-79 | 0.01629342520815389 | 0.01760126927462938 |
| 80-85 | 0.013879003558718862 | 0.019004304393730204 |
| >85 | 0.010060240963855422 | 0.01336603310852238 |
| | None | None |
| 0-4 | 0.018728641307989548 | 0.03319186463098716 |
| 5-9 | 0.01028075650432146 | 0.014004627029940927 |
| 10-14 | 0.009477653230598987 | 0.009537410108634672 |
| 15-19 | 0.008035776675284746 | 0.00490154332377627 |
| 20-24 | 0.007115362183599674 | 0.004500402759780058 |
| 25-29 | 0.007577821011673151 | 0.005248203532979398 |
| 30-34 | 0.006884123609111452 | 0.004766477218661061 |
| 35-39 | 0.0065546766416719575 | 0.004349323572052192 |
| 40-44 | 0.006626081587092995 | 0.003930189181987743 |
| 45-49 | 0.006418246916854529 | 0.003756154092728513 |
| 50-54 | 0.006376360808709175 | 0.004200451391791356 |
| 55-59 | 0.006979787698124182 | 0.004478087870343412 |
| 60-64 | 0.007170134829709298 | 0.005344440640572102 |
| 65-69 | 0.0072567619827566595 | 0.006549966558269333 |
| 70-74 | 0.00796138098367285 | 0.007485520708119331 |
| 75-79 | 0.008127578351086755 | 0.009503154574132491 |
| 80-85 | 0.007580778790389395 | 0.008312228066757582 |
| >85 | 0.0064516129032258064 | 0.008215381569830744 |
| | None | None |
| 0-4 | 0.013559624832514515 | 0.023500443732924984 |
| 5-9 | 0.010348642960970213 | 0.015502006270967176 |
| 10-14 | 0.007640980344163376 | 0.008478819278157516 |
| 15-19 | 0.0070921985815602835 | 0.004351711543691961 |
| 20-24 | 0.005753472424013483 | 0.0033898838315912006 |
| 25-29 | 0.005692472041894204 | 0.0037582041732288785 |
| 30-34 | 0.005337214944201844 | 0.004178964902539525 |
| 35-39 | 0.005872211126474454 | 0.003842575433235252 |
| 40-44 | 0.005813604520549119 | 0.0036909713163185438 |
| 45-49 | 0.005728815636296796 | 0.003471619510501649 |
| 50-54 | 0.004968549250535332 | 0.003549524857906679 |
| 55-59 | 0.00524396045884654 | 0.0037171482503267025 |
| 60-64 | 0.005601486548353217 | 0.004061030758785022 |
| 65-69 | 0.006218223742652479 | 0.004446119065561417 |
| 70-74 | 0.006133212419158532 | 0.005064730354789529 |
| 75-79 | 0.005822375752172944 | 0.004959422903516681 |
| 80-85 | 0.005091613180823981 | 0.006079177581179262 |
| >85 | 0.005142996142752893 | 0.006190372150608113 |
| | None | None |
| 0-4 | 0.012373120514648986 | 0.020651918098376087 |
| 5-9 | 0.009345954365724654 | 0.014947902290919128 |
| 10-14 | 0.007747541548090459 | 0.008456462189756786 |
| 15-19 | 0.007597217356488664 | 0.004047119314701186 |
| 20-24 | 0.005923478667133871 | 0.0035509247703171677 |
| 25-29 | 0.006292620349740058 | 0.004059479076340046 |
| 30-34 | 0.00560128678209859 | 0.0037501904393582486 |
| 35-39 | 0.005900318316079263 | 0.0039016928939932076 |
| 40-44 | 0.006268560879685368 | 0.004653582474594148 |
| 45-49 | 0.006405876323881107 | 0.0042595118993481885 |
| 50-54 | 0.006081606114908261 | 0.004748171074845244 |
| 55-59 | 0.005991248737798721 | 0.004894246624852231 |
| 60-64 | 0.006001899731741177 | 0.004567088120763425 |
| 65-69 | 0.006266942215303685 | 0.004650920413241444 |
| 70-74 | 0.006680560154256666 | 0.005349225074238295 |
| 75-79 | 0.006007702182284981 | 0.005795981452859351 |
| 80-85 | 0.005688018783689937 | 0.005522067192176369 |
| >85 | 0.006069802731411229 | 0.005304212168486739 |
Figure S1. A. Asthma prevalence and B. Asthma incidence in females and males stratified by age.

## Slide 4
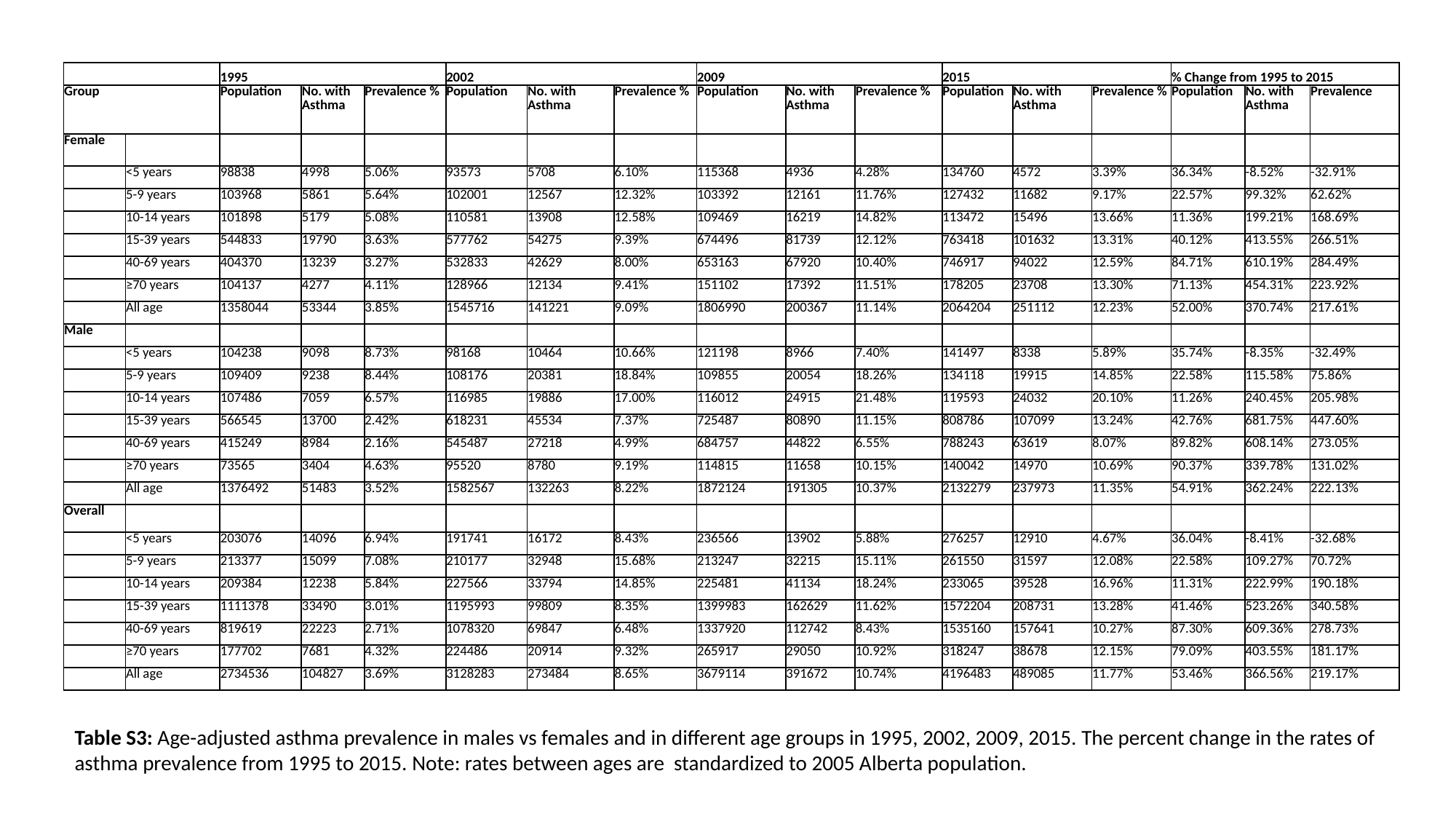

| | | 1995 | | | 2002 | | | 2009 | | | 2015 | | | % Change from 1995 to 2015 | | |
| --- | --- | --- | --- | --- | --- | --- | --- | --- | --- | --- | --- | --- | --- | --- | --- | --- |
| Group | | Population | No. with Asthma | Prevalence % | Population | No. with Asthma | Prevalence % | Population | No. with Asthma | Prevalence % | Population | No. with Asthma | Prevalence % | Population | No. with Asthma | Prevalence |
| Female | | | | | | | | | | | | | | | | |
| | <5 years | 98838 | 4998 | 5.06% | 93573 | 5708 | 6.10% | 115368 | 4936 | 4.28% | 134760 | 4572 | 3.39% | 36.34% | -8.52% | -32.91% |
| | 5-9 years | 103968 | 5861 | 5.64% | 102001 | 12567 | 12.32% | 103392 | 12161 | 11.76% | 127432 | 11682 | 9.17% | 22.57% | 99.32% | 62.62% |
| | 10-14 years | 101898 | 5179 | 5.08% | 110581 | 13908 | 12.58% | 109469 | 16219 | 14.82% | 113472 | 15496 | 13.66% | 11.36% | 199.21% | 168.69% |
| | 15-39 years | 544833 | 19790 | 3.63% | 577762 | 54275 | 9.39% | 674496 | 81739 | 12.12% | 763418 | 101632 | 13.31% | 40.12% | 413.55% | 266.51% |
| | 40-69 years | 404370 | 13239 | 3.27% | 532833 | 42629 | 8.00% | 653163 | 67920 | 10.40% | 746917 | 94022 | 12.59% | 84.71% | 610.19% | 284.49% |
| | ≥70 years | 104137 | 4277 | 4.11% | 128966 | 12134 | 9.41% | 151102 | 17392 | 11.51% | 178205 | 23708 | 13.30% | 71.13% | 454.31% | 223.92% |
| | All age | 1358044 | 53344 | 3.85% | 1545716 | 141221 | 9.09% | 1806990 | 200367 | 11.14% | 2064204 | 251112 | 12.23% | 52.00% | 370.74% | 217.61% |
| Male | | | | | | | | | | | | | | | | |
| | <5 years | 104238 | 9098 | 8.73% | 98168 | 10464 | 10.66% | 121198 | 8966 | 7.40% | 141497 | 8338 | 5.89% | 35.74% | -8.35% | -32.49% |
| | 5-9 years | 109409 | 9238 | 8.44% | 108176 | 20381 | 18.84% | 109855 | 20054 | 18.26% | 134118 | 19915 | 14.85% | 22.58% | 115.58% | 75.86% |
| | 10-14 years | 107486 | 7059 | 6.57% | 116985 | 19886 | 17.00% | 116012 | 24915 | 21.48% | 119593 | 24032 | 20.10% | 11.26% | 240.45% | 205.98% |
| | 15-39 years | 566545 | 13700 | 2.42% | 618231 | 45534 | 7.37% | 725487 | 80890 | 11.15% | 808786 | 107099 | 13.24% | 42.76% | 681.75% | 447.60% |
| | 40-69 years | 415249 | 8984 | 2.16% | 545487 | 27218 | 4.99% | 684757 | 44822 | 6.55% | 788243 | 63619 | 8.07% | 89.82% | 608.14% | 273.05% |
| | ≥70 years | 73565 | 3404 | 4.63% | 95520 | 8780 | 9.19% | 114815 | 11658 | 10.15% | 140042 | 14970 | 10.69% | 90.37% | 339.78% | 131.02% |
| | All age | 1376492 | 51483 | 3.52% | 1582567 | 132263 | 8.22% | 1872124 | 191305 | 10.37% | 2132279 | 237973 | 11.35% | 54.91% | 362.24% | 222.13% |
| Overall | | | | | | | | | | | | | | | | |
| | <5 years | 203076 | 14096 | 6.94% | 191741 | 16172 | 8.43% | 236566 | 13902 | 5.88% | 276257 | 12910 | 4.67% | 36.04% | -8.41% | -32.68% |
| | 5-9 years | 213377 | 15099 | 7.08% | 210177 | 32948 | 15.68% | 213247 | 32215 | 15.11% | 261550 | 31597 | 12.08% | 22.58% | 109.27% | 70.72% |
| | 10-14 years | 209384 | 12238 | 5.84% | 227566 | 33794 | 14.85% | 225481 | 41134 | 18.24% | 233065 | 39528 | 16.96% | 11.31% | 222.99% | 190.18% |
| | 15-39 years | 1111378 | 33490 | 3.01% | 1195993 | 99809 | 8.35% | 1399983 | 162629 | 11.62% | 1572204 | 208731 | 13.28% | 41.46% | 523.26% | 340.58% |
| | 40-69 years | 819619 | 22223 | 2.71% | 1078320 | 69847 | 6.48% | 1337920 | 112742 | 8.43% | 1535160 | 157641 | 10.27% | 87.30% | 609.36% | 278.73% |
| | ≥70 years | 177702 | 7681 | 4.32% | 224486 | 20914 | 9.32% | 265917 | 29050 | 10.92% | 318247 | 38678 | 12.15% | 79.09% | 403.55% | 181.17% |
| | All age | 2734536 | 104827 | 3.69% | 3128283 | 273484 | 8.65% | 3679114 | 391672 | 10.74% | 4196483 | 489085 | 11.77% | 53.46% | 366.56% | 219.17% |
Table S3: Age-adjusted asthma prevalence in males vs females and in different age groups in 1995, 2002, 2009, 2015. The percent change in the rates of asthma prevalence from 1995 to 2015. Note: rates between ages are standardized to 2005 Alberta population.

## Slide 5
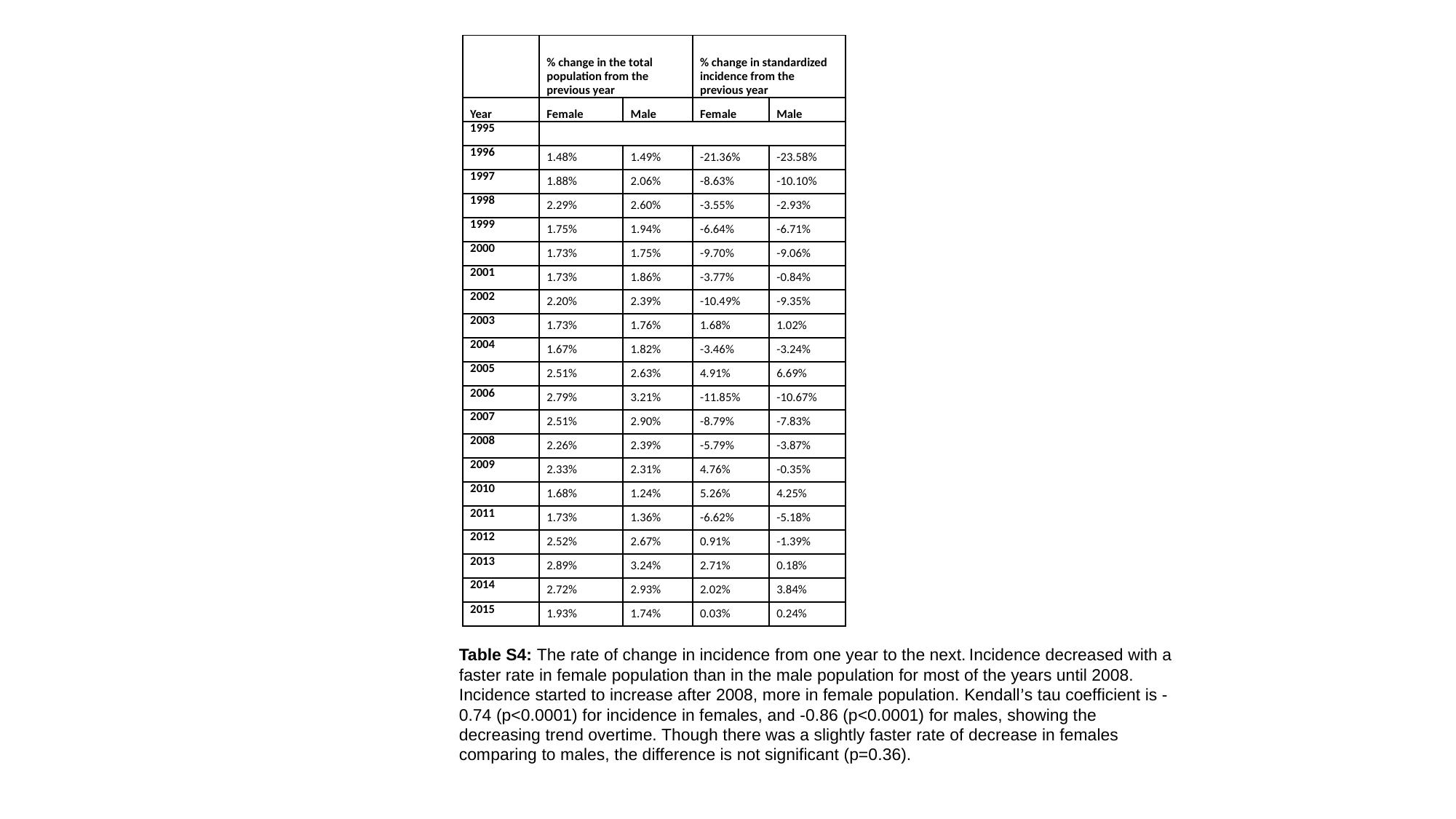

| | % change in the total population from the previous year | | % change in standardized incidence from the previous year | |
| --- | --- | --- | --- | --- |
| Year | Female | Male | Female | Male |
| 1995 | | | | |
| 1996 | 1.48% | 1.49% | -21.36% | -23.58% |
| 1997 | 1.88% | 2.06% | -8.63% | -10.10% |
| 1998 | 2.29% | 2.60% | -3.55% | -2.93% |
| 1999 | 1.75% | 1.94% | -6.64% | -6.71% |
| 2000 | 1.73% | 1.75% | -9.70% | -9.06% |
| 2001 | 1.73% | 1.86% | -3.77% | -0.84% |
| 2002 | 2.20% | 2.39% | -10.49% | -9.35% |
| 2003 | 1.73% | 1.76% | 1.68% | 1.02% |
| 2004 | 1.67% | 1.82% | -3.46% | -3.24% |
| 2005 | 2.51% | 2.63% | 4.91% | 6.69% |
| 2006 | 2.79% | 3.21% | -11.85% | -10.67% |
| 2007 | 2.51% | 2.90% | -8.79% | -7.83% |
| 2008 | 2.26% | 2.39% | -5.79% | -3.87% |
| 2009 | 2.33% | 2.31% | 4.76% | -0.35% |
| 2010 | 1.68% | 1.24% | 5.26% | 4.25% |
| 2011 | 1.73% | 1.36% | -6.62% | -5.18% |
| 2012 | 2.52% | 2.67% | 0.91% | -1.39% |
| 2013 | 2.89% | 3.24% | 2.71% | 0.18% |
| 2014 | 2.72% | 2.93% | 2.02% | 3.84% |
| 2015 | 1.93% | 1.74% | 0.03% | 0.24% |
Table S4: The rate of change in incidence from one year to the next. Incidence decreased with a faster rate in female population than in the male population for most of the years until 2008. Incidence started to increase after 2008, more in female population. Kendall’s tau coefficient is -0.74 (p<0.0001) for incidence in females, and -0.86 (p<0.0001) for males, showing the decreasing trend overtime. Though there was a slightly faster rate of decrease in females comparing to males, the difference is not significant (p=0.36).

## Slide 6
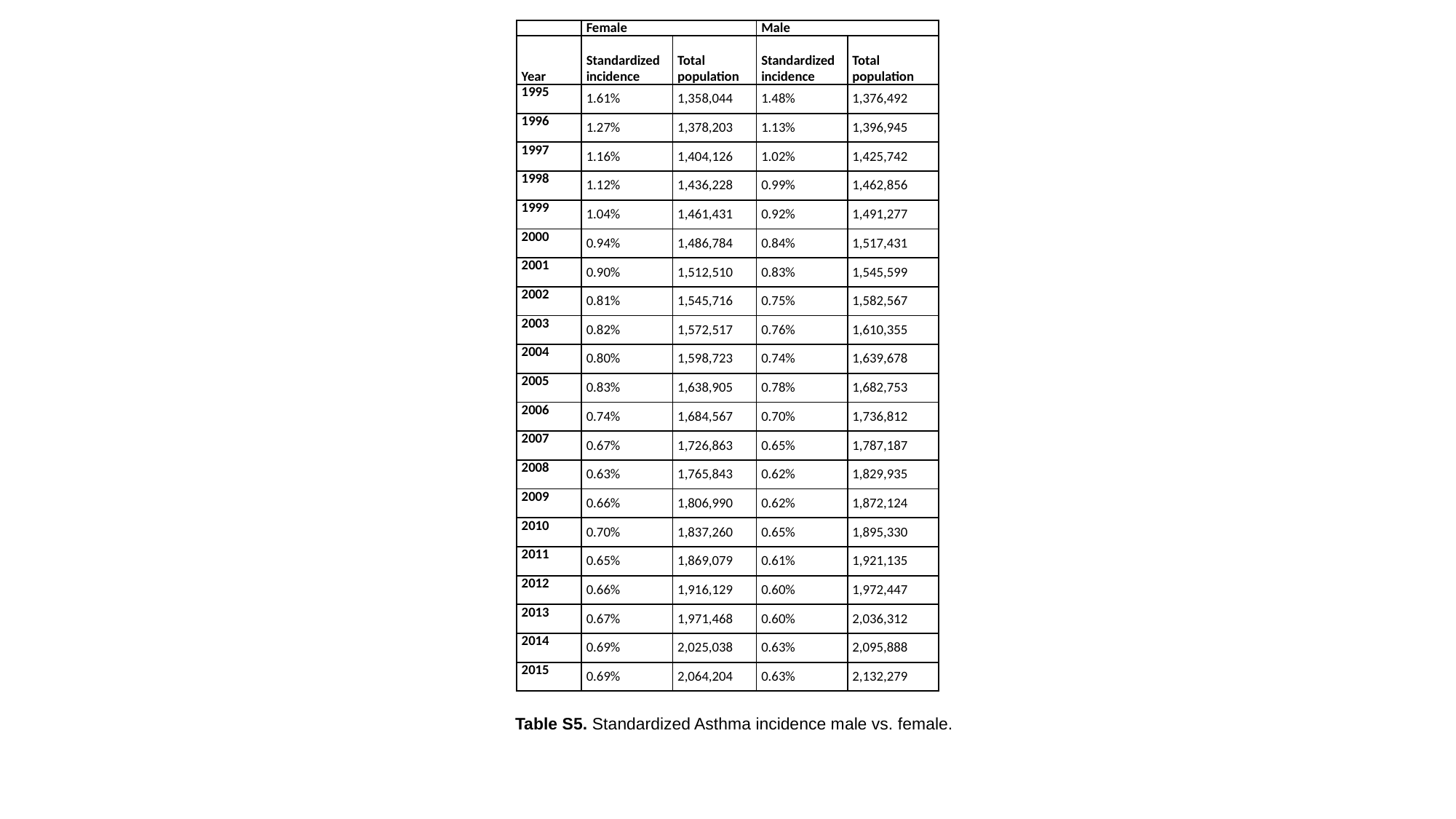

| | Female | | Male | |
| --- | --- | --- | --- | --- |
| Year | Standardized incidence | Total population | Standardized incidence | Total population |
| 1995 | 1.61% | 1,358,044 | 1.48% | 1,376,492 |
| 1996 | 1.27% | 1,378,203 | 1.13% | 1,396,945 |
| 1997 | 1.16% | 1,404,126 | 1.02% | 1,425,742 |
| 1998 | 1.12% | 1,436,228 | 0.99% | 1,462,856 |
| 1999 | 1.04% | 1,461,431 | 0.92% | 1,491,277 |
| 2000 | 0.94% | 1,486,784 | 0.84% | 1,517,431 |
| 2001 | 0.90% | 1,512,510 | 0.83% | 1,545,599 |
| 2002 | 0.81% | 1,545,716 | 0.75% | 1,582,567 |
| 2003 | 0.82% | 1,572,517 | 0.76% | 1,610,355 |
| 2004 | 0.80% | 1,598,723 | 0.74% | 1,639,678 |
| 2005 | 0.83% | 1,638,905 | 0.78% | 1,682,753 |
| 2006 | 0.74% | 1,684,567 | 0.70% | 1,736,812 |
| 2007 | 0.67% | 1,726,863 | 0.65% | 1,787,187 |
| 2008 | 0.63% | 1,765,843 | 0.62% | 1,829,935 |
| 2009 | 0.66% | 1,806,990 | 0.62% | 1,872,124 |
| 2010 | 0.70% | 1,837,260 | 0.65% | 1,895,330 |
| 2011 | 0.65% | 1,869,079 | 0.61% | 1,921,135 |
| 2012 | 0.66% | 1,916,129 | 0.60% | 1,972,447 |
| 2013 | 0.67% | 1,971,468 | 0.60% | 2,036,312 |
| 2014 | 0.69% | 2,025,038 | 0.63% | 2,095,888 |
| 2015 | 0.69% | 2,064,204 | 0.63% | 2,132,279 |
Table S5. Standardized Asthma incidence male vs. female.

## Slide 7
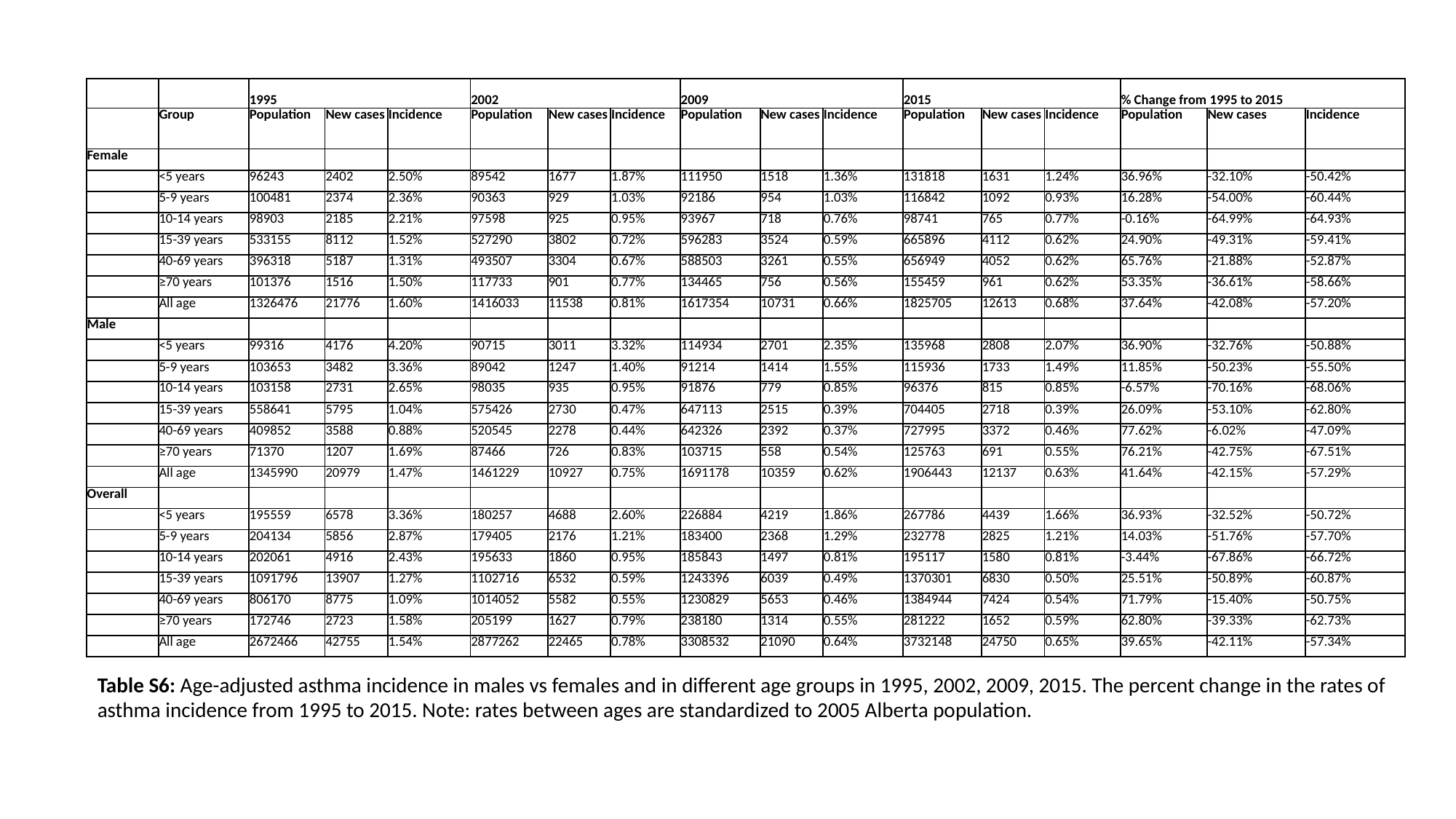

| | | 1995 | | | 2002 | | | 2009 | | | 2015 | | | % Change from 1995 to 2015 | | |
| --- | --- | --- | --- | --- | --- | --- | --- | --- | --- | --- | --- | --- | --- | --- | --- | --- |
| | Group | Population | New cases | Incidence | Population | New cases | Incidence | Population | New cases | Incidence | Population | New cases | Incidence | Population | New cases | Incidence |
| Female | | | | | | | | | | | | | | | | |
| | <5 years | 96243 | 2402 | 2.50% | 89542 | 1677 | 1.87% | 111950 | 1518 | 1.36% | 131818 | 1631 | 1.24% | 36.96% | -32.10% | -50.42% |
| | 5-9 years | 100481 | 2374 | 2.36% | 90363 | 929 | 1.03% | 92186 | 954 | 1.03% | 116842 | 1092 | 0.93% | 16.28% | -54.00% | -60.44% |
| | 10-14 years | 98903 | 2185 | 2.21% | 97598 | 925 | 0.95% | 93967 | 718 | 0.76% | 98741 | 765 | 0.77% | -0.16% | -64.99% | -64.93% |
| | 15-39 years | 533155 | 8112 | 1.52% | 527290 | 3802 | 0.72% | 596283 | 3524 | 0.59% | 665896 | 4112 | 0.62% | 24.90% | -49.31% | -59.41% |
| | 40-69 years | 396318 | 5187 | 1.31% | 493507 | 3304 | 0.67% | 588503 | 3261 | 0.55% | 656949 | 4052 | 0.62% | 65.76% | -21.88% | -52.87% |
| | ≥70 years | 101376 | 1516 | 1.50% | 117733 | 901 | 0.77% | 134465 | 756 | 0.56% | 155459 | 961 | 0.62% | 53.35% | -36.61% | -58.66% |
| | All age | 1326476 | 21776 | 1.60% | 1416033 | 11538 | 0.81% | 1617354 | 10731 | 0.66% | 1825705 | 12613 | 0.68% | 37.64% | -42.08% | -57.20% |
| Male | | | | | | | | | | | | | | | | |
| | <5 years | 99316 | 4176 | 4.20% | 90715 | 3011 | 3.32% | 114934 | 2701 | 2.35% | 135968 | 2808 | 2.07% | 36.90% | -32.76% | -50.88% |
| | 5-9 years | 103653 | 3482 | 3.36% | 89042 | 1247 | 1.40% | 91214 | 1414 | 1.55% | 115936 | 1733 | 1.49% | 11.85% | -50.23% | -55.50% |
| | 10-14 years | 103158 | 2731 | 2.65% | 98035 | 935 | 0.95% | 91876 | 779 | 0.85% | 96376 | 815 | 0.85% | -6.57% | -70.16% | -68.06% |
| | 15-39 years | 558641 | 5795 | 1.04% | 575426 | 2730 | 0.47% | 647113 | 2515 | 0.39% | 704405 | 2718 | 0.39% | 26.09% | -53.10% | -62.80% |
| | 40-69 years | 409852 | 3588 | 0.88% | 520545 | 2278 | 0.44% | 642326 | 2392 | 0.37% | 727995 | 3372 | 0.46% | 77.62% | -6.02% | -47.09% |
| | ≥70 years | 71370 | 1207 | 1.69% | 87466 | 726 | 0.83% | 103715 | 558 | 0.54% | 125763 | 691 | 0.55% | 76.21% | -42.75% | -67.51% |
| | All age | 1345990 | 20979 | 1.47% | 1461229 | 10927 | 0.75% | 1691178 | 10359 | 0.62% | 1906443 | 12137 | 0.63% | 41.64% | -42.15% | -57.29% |
| Overall | | | | | | | | | | | | | | | | |
| | <5 years | 195559 | 6578 | 3.36% | 180257 | 4688 | 2.60% | 226884 | 4219 | 1.86% | 267786 | 4439 | 1.66% | 36.93% | -32.52% | -50.72% |
| | 5-9 years | 204134 | 5856 | 2.87% | 179405 | 2176 | 1.21% | 183400 | 2368 | 1.29% | 232778 | 2825 | 1.21% | 14.03% | -51.76% | -57.70% |
| | 10-14 years | 202061 | 4916 | 2.43% | 195633 | 1860 | 0.95% | 185843 | 1497 | 0.81% | 195117 | 1580 | 0.81% | -3.44% | -67.86% | -66.72% |
| | 15-39 years | 1091796 | 13907 | 1.27% | 1102716 | 6532 | 0.59% | 1243396 | 6039 | 0.49% | 1370301 | 6830 | 0.50% | 25.51% | -50.89% | -60.87% |
| | 40-69 years | 806170 | 8775 | 1.09% | 1014052 | 5582 | 0.55% | 1230829 | 5653 | 0.46% | 1384944 | 7424 | 0.54% | 71.79% | -15.40% | -50.75% |
| | ≥70 years | 172746 | 2723 | 1.58% | 205199 | 1627 | 0.79% | 238180 | 1314 | 0.55% | 281222 | 1652 | 0.59% | 62.80% | -39.33% | -62.73% |
| | All age | 2672466 | 42755 | 1.54% | 2877262 | 22465 | 0.78% | 3308532 | 21090 | 0.64% | 3732148 | 24750 | 0.65% | 39.65% | -42.11% | -57.34% |
Table S6: Age-adjusted asthma incidence in males vs females and in different age groups in 1995, 2002, 2009, 2015. The percent change in the rates of asthma incidence from 1995 to 2015. Note: rates between ages are standardized to 2005 Alberta population.

## Slide 8
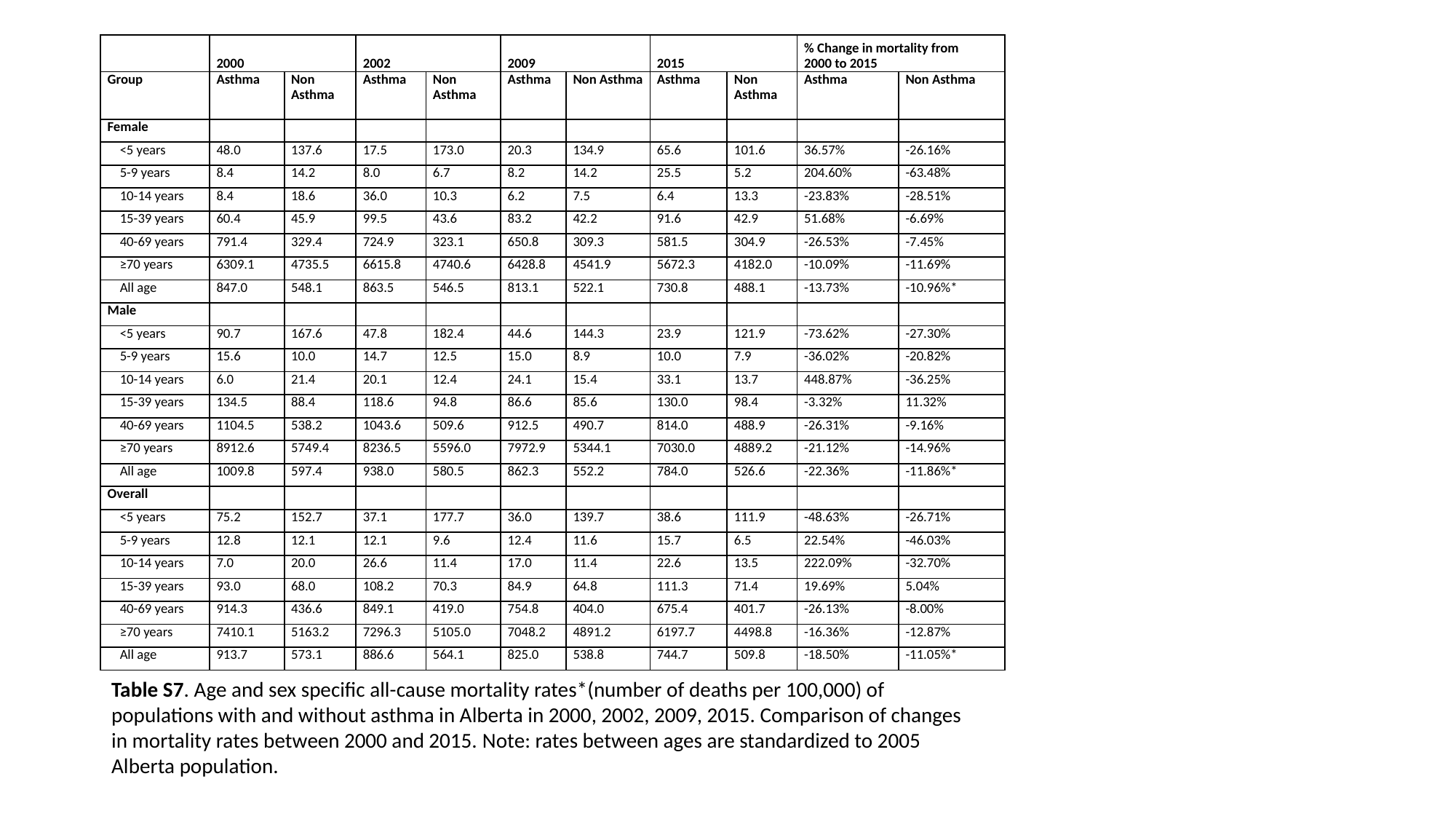

| | 2000 | | 2002 | | 2009 | | 2015 | | % Change in mortality from 2000 to 2015 | |
| --- | --- | --- | --- | --- | --- | --- | --- | --- | --- | --- |
| Group | Asthma | Non Asthma | Asthma | Non Asthma | Asthma | Non Asthma | Asthma | Non Asthma | Asthma | Non Asthma |
| Female | | | | | | | | | | |
| <5 years | 48.0 | 137.6 | 17.5 | 173.0 | 20.3 | 134.9 | 65.6 | 101.6 | 36.57% | -26.16% |
| 5-9 years | 8.4 | 14.2 | 8.0 | 6.7 | 8.2 | 14.2 | 25.5 | 5.2 | 204.60% | -63.48% |
| 10-14 years | 8.4 | 18.6 | 36.0 | 10.3 | 6.2 | 7.5 | 6.4 | 13.3 | -23.83% | -28.51% |
| 15-39 years | 60.4 | 45.9 | 99.5 | 43.6 | 83.2 | 42.2 | 91.6 | 42.9 | 51.68% | -6.69% |
| 40-69 years | 791.4 | 329.4 | 724.9 | 323.1 | 650.8 | 309.3 | 581.5 | 304.9 | -26.53% | -7.45% |
| ≥70 years | 6309.1 | 4735.5 | 6615.8 | 4740.6 | 6428.8 | 4541.9 | 5672.3 | 4182.0 | -10.09% | -11.69% |
| All age | 847.0 | 548.1 | 863.5 | 546.5 | 813.1 | 522.1 | 730.8 | 488.1 | -13.73% | -10.96%\* |
| Male | | | | | | | | | | |
| <5 years | 90.7 | 167.6 | 47.8 | 182.4 | 44.6 | 144.3 | 23.9 | 121.9 | -73.62% | -27.30% |
| 5-9 years | 15.6 | 10.0 | 14.7 | 12.5 | 15.0 | 8.9 | 10.0 | 7.9 | -36.02% | -20.82% |
| 10-14 years | 6.0 | 21.4 | 20.1 | 12.4 | 24.1 | 15.4 | 33.1 | 13.7 | 448.87% | -36.25% |
| 15-39 years | 134.5 | 88.4 | 118.6 | 94.8 | 86.6 | 85.6 | 130.0 | 98.4 | -3.32% | 11.32% |
| 40-69 years | 1104.5 | 538.2 | 1043.6 | 509.6 | 912.5 | 490.7 | 814.0 | 488.9 | -26.31% | -9.16% |
| ≥70 years | 8912.6 | 5749.4 | 8236.5 | 5596.0 | 7972.9 | 5344.1 | 7030.0 | 4889.2 | -21.12% | -14.96% |
| All age | 1009.8 | 597.4 | 938.0 | 580.5 | 862.3 | 552.2 | 784.0 | 526.6 | -22.36% | -11.86%\* |
| Overall | | | | | | | | | | |
| <5 years | 75.2 | 152.7 | 37.1 | 177.7 | 36.0 | 139.7 | 38.6 | 111.9 | -48.63% | -26.71% |
| 5-9 years | 12.8 | 12.1 | 12.1 | 9.6 | 12.4 | 11.6 | 15.7 | 6.5 | 22.54% | -46.03% |
| 10-14 years | 7.0 | 20.0 | 26.6 | 11.4 | 17.0 | 11.4 | 22.6 | 13.5 | 222.09% | -32.70% |
| 15-39 years | 93.0 | 68.0 | 108.2 | 70.3 | 84.9 | 64.8 | 111.3 | 71.4 | 19.69% | 5.04% |
| 40-69 years | 914.3 | 436.6 | 849.1 | 419.0 | 754.8 | 404.0 | 675.4 | 401.7 | -26.13% | -8.00% |
| ≥70 years | 7410.1 | 5163.2 | 7296.3 | 5105.0 | 7048.2 | 4891.2 | 6197.7 | 4498.8 | -16.36% | -12.87% |
| All age | 913.7 | 573.1 | 886.6 | 564.1 | 825.0 | 538.8 | 744.7 | 509.8 | -18.50% | -11.05%\* |
Table S7. Age and sex specific all-cause mortality rates*(number of deaths per 100,000) of populations with and without asthma in Alberta in 2000, 2002, 2009, 2015. Comparison of changes in mortality rates between 2000 and 2015. Note: rates between ages are standardized to 2005 Alberta population.
